# Immune dysregulation in patients with PTEN hamartoma tumor syndrome: Analysis of FOXP3 regulatory T cells

**DOI:** 10.1016/j.jaci.2016.03.059

**Published:** 2017-02

**Authors:** Hannah H. Chen, Norman Händel, Joanne Ngeow, James Muller, Michael Hühn, Huei-Ting Yang, Mario Heindl, Roos-Marijn Berbers, Ahmed N. Hegazy, Janina Kionke, Lamis Yehia, Ulrich Sack, Frank Bläser, Anne Rensing-Ehl, Julia Reifenberger, Julia Keith, Simon Travis, Andreas Merkenschlager, Wieland Kiess, Christian Wittekind, Lisa Walker, Stephan Ehl, Stefan Aretz, Michael L. Dustin, Charis Eng, Fiona Powrie, Holm H. Uhlig

**Affiliations:** a Translational Gastroenterology Unit, University of Oxford, Oxford, United Kingdom; l Kennedy Institute of Rheumatology, University of Oxford, Oxford, United Kingdom; n Department of Pediatrics, University of Oxford, Oxford, United Kingdom; b Hospital for Children and Adolescents, University of Leipzig, Leipzig, Germany; f Department of Internal Medicine, University of Leipzig, Leipzig, Germany; g Institute for Immunology and Transfusion Medicine, University of Leipzig, Leipzig, Germany; j Institute for Pathology, University of Leipzig, Leipzig, Germany; c Genomic Medicine Institute, Lerner Research Institute, and Taussig Cancer Institute, Cleveland Clinic, Cleveland, Ohio; d Institute of Human Genetics, Biomedical Center, University of Bonn, Bonn, Germany; e Skirball Institute of Biomolecular Medicine, New York University School of Medicine, New York, NY; h Centre of Chronic Immunodeficiency, University Medical Centre, Freiburg, Germany; i Department of Dermatology, Heinrich-Heine-University, Dusseldorf, Germany; k Department of Clinical Genetics, Churchill Hospital, Oxford, United Kingdom; m Department of Genetics and Genome Sciences, and CASE Comprehensive Cancer Center, Case Western Reserve University School of Medicine, Cleveland, Ohio

**Keywords:** Regulatory T cells, phosphatases, phosphoinositide 3-kinase, PTEN, PH domain leucine-rich repeat protein phosphatase, PHTS, immunologic synapse, autoimmunity, APC, Allophycocyanin, CS, Cowden syndrome, CTLA-4, Cytotoxic T lymphocyte–associated antigen 4, DLG1, Scaffold protein discs, large homolog 1, FACS, Fluorescence-activated cell sorting, FITC, Fluorescein isothiocyanate, FOXP3, Forkhead box P3, HRM, High Resolution DNA Melting, IC_50_, Inhibitory concentration of 50%, ICAM-1, Intercellular adhesion molecule 1, iTreg, *In vitro* induced regulatory T, MALT, Mucosa-associated lymphoid tissue, mTOR, Mammalian target of rapamycin, mTORC1, PTEN/AKT/mTOR complex 1, NHERF1, Na^+^/H^+^-exchanger 3 regulatory factor, PE, Phycoerythrin, PerCP, Peridinin-chlorophyll-protein complex, PHLPP, PH domain leucine-rich repeat protein phosphatase, PHTS, *PTEN* hamartoma tumor syndrome, PI3K, Phosphoinositide 3-kinase, POD, Peroxidase, PP2A, Protein phosphatase 2A, PTEN, Phosphatase and tensin homologue deleted on chromosome 10, SHIP, Src homology domain 2–containing inositol phosphatase, TCR, T-cell receptor, Tmem, Memory T, TMRE, Tetramethylrhodamine-ethylester

## Abstract

**Background:**

Patients with heterozygous germline mutations in phosphatase and tensin homolog deleted on chromosome 10 *(PTEN)* experience autoimmunity and lymphoid hyperplasia.

**Objectives:**

Because regulation of the phosphoinositide 3-kinase (PI3K) pathway is critical for maintaining regulatory T (Treg) cell functions, we investigate Treg cells in patients with heterozygous germline PTEN mutations (PTEN hamartoma tumor syndrome [PHTS]).

**Methods:**

Patients with PHTS were assessed for immunologic conditions, lymphocyte subsets, forkhead box P3 (FOXP3)^+^ Treg cell levels, and phenotype. To determine the functional importance of phosphatases that control the PI3K pathway, we assessed Treg cell induction *in vitro*, mitochondrial depolarization, and recruitment of PTEN to the immunologic synapse.

**Results:**

Autoimmunity and peripheral lymphoid hyperplasia were found in 43% of 79 patients with PHTS. Immune dysregulation in patients with PHTS included lymphopenia, CD4^+^ T-cell reduction, and changes in T- and B-cell subsets. Although total CD4^+^FOXP3^+^ Treg cell numbers are reduced, frequencies are maintained in the blood and intestine. Despite pathogenic PTEN mutations, the FOXP3^+^ T cells are phenotypically normal. We show that the phosphatase PH domain leucine-rich repeat protein phosphatase (PHLPP) downstream of PTEN is highly expressed in normal human Treg cells and provides complementary phosphatase activity. PHLPP is indispensable for the differentiation of induced Treg cells *in vitro* and Treg cell mitochondrial fitness. PTEN and PHLPP form a phosphatase network that is polarized at the immunologic synapse.

**Conclusion:**

Heterozygous loss of function of PTEN in human subjects has a significant effect on T- and B-cell immunity. Assembly of the PTEN-PHLPP phosphatase network allows coordinated phosphatase activities at the site of T-cell receptor activation, which is important for limiting PI3K hyperactivation in Treg cells despite PTEN haploinsufficiency.

## Graphical abstract

**Figure:**
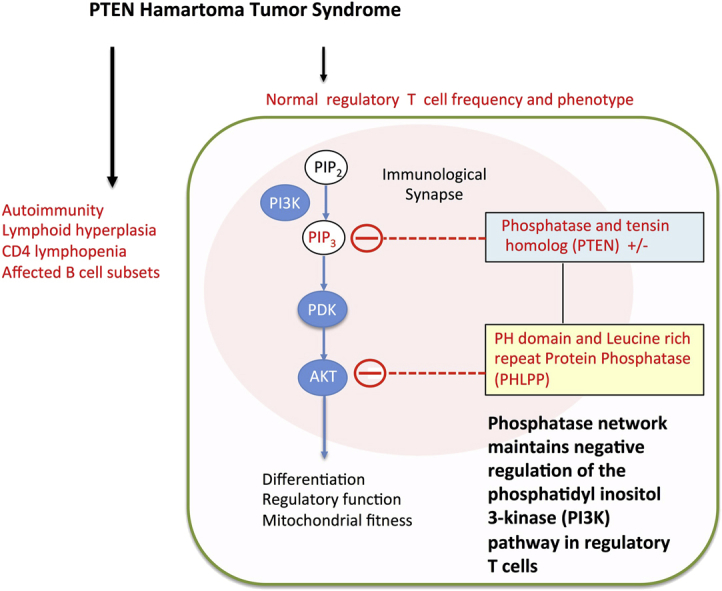

Generation of the second messenger phosphatidylinositol-3,4,5-trisphosphate by phosphoinositide 3-kinase (PI3K) constitutes a critical checkpoint for immune activation.^1^ This pathway is controlled by phosphatases, such as PTEN, a dual-specific protein and lipid phosphatase. *Pten* deletion in immune cell subsets in mice caused defects in T cells,^2^, ^3^ CD4^+^Foxp3^+^ regulatory T (Treg) cells^4^, ^5^, ^6^ and B cells.^7^ Heterozygous *Pten* deletion caused autoimmunity, intestinal lymphoid hyperplasia, thymus hyperplasia, and thymoma and T-cell lymphoma formation.^8^, ^9^

Heterozygous PTEN mutations are found in a group of hereditary disorders known as *PTEN* hamartoma tumor syndrome (PHTS).^10^ Patients with PHTS can present with autoimmunity, lymphoid hyperplasia, colitis and lymphopenia, as well as defects in B cell responses^11^, ^12^ and low immunoglobulin levels.^11^, ^13^

The PI3K/AKT/mammalian target of rapamycin (mTOR) signaling pathway is pivotal for Treg cell development and homeostasis.^5^, ^6^, ^14^, ^15^, ^16^, ^17^, ^18^ This pathway is activated downstream of the T-cell receptor (TCR), CD28, and IL-2 signaling. It is critically involved in Treg cell thymic development, peripheral expansion, and suppressive activity.^18^ Constitutively active Akt impairs CD4^+^Foxp3^+^ T-cell differentiation in the thymus but does not affect established Foxp3 expression in Treg cells.^14^ Akt inhibits the FoxO family of transcription factors, FoxO1 and FoxO3a, which direct both Foxp3-dependent and independent suppressive programs in Treg cells.^19^, ^20^, ^21^, ^22^ The metabolic checkpoint kinase mTOR orchestrates Treg cell metabolic programs and suppressive function.^23^, ^24^ Although mTOR activity is critical for differentiation into T_H_1 and T_H_2 lineages and T_H_17 lineage commitment, TCR engagement in the absence of mTOR leads to Treg cell differentiation.^17^ These observations highlight the importance of a stringent negative regulation of PI3K pathway activity in Treg cells.

We describe immune dysregulation in patients with PHTS. We expected that because of increased PI3K/AKT signaling, Treg cell generation and stability would be affected. However, we detected no abnormal accumulation of these cells. Instead, we identified a phosphatase network in which the phosphatase PH domain leucine-rich repeat protein phosphatase (PHLPP) acts as an essential phosphatase downstream of PTEN, thereby preventing excessive AKT activation in Treg cells, and provides functional complementation for PTEN. We show that PTEN and PHLPP act to sustain mitochondrial metabolism in Treg cells. PTEN and PHLPP form a phosphatase network supported by the scaffold protein Na^+^/H^+^-exchanger 3 regulatory factor (NHERF1), allowing polarization of phosphatase activity toward the immunologic synapse in Treg cells. This polarized network might allow maintenance of Treg cell function through coordinated phosphatase activities to restrain phospho-AKT accumulation.

## Methods

### Patients, material, and clinical methods

**Fig 1:**
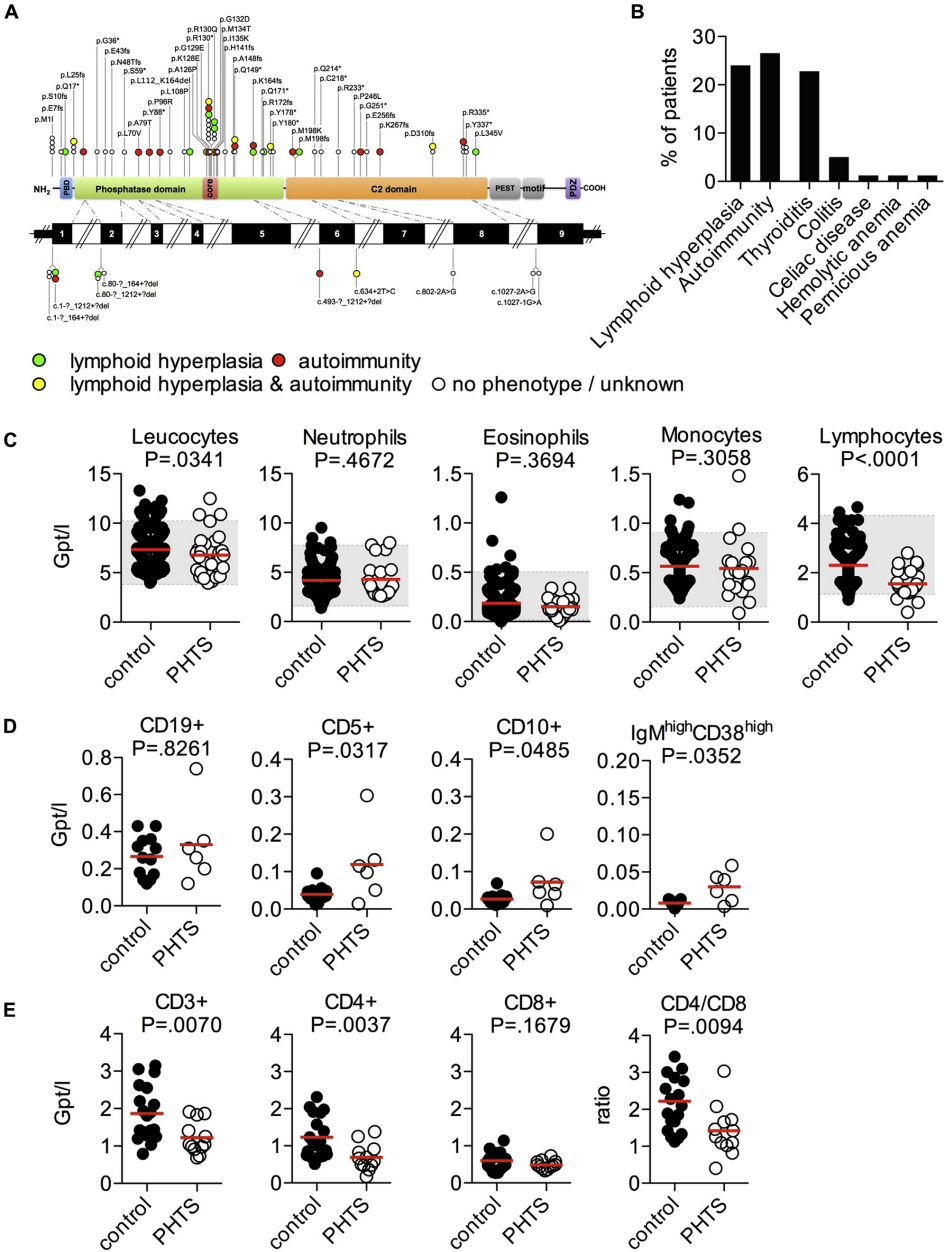
PHTS patient cohort and clinical phenotype. **A,** Representation of different pathogenic germline *PTEN* mutations in 79 patients with PHTS investigated. *Symbols* represent the mutation site of individual patients. *Colored symbols* represent patients who present with autoimmunity, lymphoid hyperplasia, or both. **B,** Immunologic conditions in the PHTS patient cohort. **C,** Peripheral blood leukocyte counts of adult patients with PHTS (n = 32) and blood donor control subjects (n = 216). Each *dot* represents 1 patient. *Gray boxes* mark the normal range. Statistical differences were analyzed by using the Mann-Whitney test. **D,** Numbers of CD19^+^, CD5^+^, CD10^+^ immature, and IgM^high^CD38^high^ transitional B cells. **E,** Numbers of CD3^+^ T cells, percentages of CD4^+^ and CD8^+^ T cells among CD3^+^ T cells, and CD4^+^/CD8^+^ T-cell ratio.

Seventy-nine patients with pathogenic germline *PTEN* mutations were enrolled in the study (39 male and 40 female patients; Fig 1, *A*, and see Tables E1 and E2 in this article's Online Repository at www.jacionline.org). Ethical review was granted by the institutional ethical review boards (Leipzig, Bonn, Freiburg and Oxford Universities and the Cleveland Clinic). Control blood samples were obtained from healthy volunteer donors or as leukocyte cones from a United Kingdom blood donor bank. Patients' biopsy specimens were derived from routine endoscopies and noninflammatory mucosa-associated lymphoid tissue (MALT) control tissues after appendectomy (n = 10). Written informed consent was obtained for all patients and control subjects.

### Immunohistochemistry and fluorescence microscopy

Paraffin-embedded biopsy specimens were used for immunohistochemistry by using multicolor fluorescence staining and tyramide amplification, essentially as described previously^11^ and specified in the Methods section in this article's Online Repository at www.jacionline.org. Fluorescence images were recorded with a Keyence BZ-8000 (Keyence, Osaka, Japan) or Zeiss Axioscope (Zeiss, Oberkochen, Germany) fluorescence microscope.

### Flow cytometry

Leukocyte subsets from patients with PHTS and healthy control subjects were analyzed by using fluorescence-activated cell sorting (FACS; as specified in the Methods section in this article's Online Repository). Treg cells from blood were detected by means of intracellular staining for forkhead box P3 (FOXP3; Foxp3/Transcription Factor Staining Buffer Set; eBioscience, San Diego, Calif) and/or the cell-surface markers CD127, CD25, and CD4, as indicated. Intracellular staining was performed for PTEN (clone Y184; Epitomics, Burlingame, Calif), cytotoxic T lymphocyte–associated antigen 4 (CTLA-4; clone 14D3, eBioscience), and Helios (clone 22F6; Miltenyi Biotec, Bergisch Gladbach, Germany).

Phosflow was performed on PBMCs from healthy donors after preincubation for 5 minutes with biotinylated anti-CD3 (clone OKT3; BioLegend, San Diego, Calif) and anti-CD28 (clone CD28.2, eBioscience; each at 3.3 μg/mL in 150 μL) and activation by avidin (150 μL of 50 μg/mL; Invitrogen, Carlsbad, Calif) and IL-2 (500 IU/mL; PeproTech, Rocky Hill, NJ). Cells were stained 10 minutes after activation with anti-phosphorylated signal transducer and activator of transcription 5 (pY694, clone 47), anti-pAKT (pS473, clone M-89-61), and anti-pS6 (pS235/p236, clone N7-548) by using BD Biosciences Phosflow reagents and analyzed on the LSR II instrument (BD Biosciences, San Jose, Calif). All FACS analyses were performed with FlowJo software (TreeStar, Ashland, Ore).

### Tetramethylrhodamine-ethylester assay

Fresh PBMCs were cultured for 20 hours in the presence of low (0.4 μmol/L), medium (2 μmol/L; inhibitory concentration of 50% [IC_50_]), or high (4 μmol/L) concentrations of the PTEN inhibitor SF1670 (Cellagen Technology, San Diego, Calif) alone or in combination with the PHLPP inhibitor (US NCI, NCS 45586; IC_50_, 70 μmol/L) to investigate the effects of PTEN/PHLPP inhibitors. Cells were then stimulated with T-cell activator beads (1 bead to 1 CD4^+^ T cell for 90 minutes). The tetramethylrhodamine-ethylester (TMRE; ab113852; Abcam, Cambridge, United Kingdom) assay was performed, according to the manufacturer's protocol. TMRE staining was combined with CD4^+^, CD25, and CD127 surface staining.

PBMCs were cultured for 20 hours and stained with indicated surface marker antibodies and TMRE to measure mitochondrial membrane potential to compare control subjects with patients with PHTS.

### PTEN, PHLPP, and NHERF1 recruitment to the immunologic synapse

Preparation of supported planar bilayer was described previously.^25^, ^26^, ^27^ The bilayers were then blocked with casein and incubated with streptavidin (5 μg/mL), monobiotinylated OKT3 labeled with Alexa Flour 647 (4 μg/mL), ICAM1-12xHis labeled with Alexa Fluor 405 (200 mol/μm^2^). CD4^+^CD25^+^ T cells (1 × 10^6^) were injected into prewarmed chambers. Anti-PHLPP (against peptides 1596-1610; Sigma, St Louis, Mo), anti-NHERF1 (against a peptide around alanine 310; Cell Signaling, Danvers, Mass), and anti-PTEN (D4.3, Cell Signaling) were detected by using donkey anti-rabbit F(ab′)_2_ fragments labeled with Alexa Fluor 488 (Jackson Laboratories, Bar Harbor, Me). CD4^+^CD25^+^ cells were rested overnight before analysis. LabTek II chambers (Thermo Scientific, Waltham, Mass) were coated with OKT3 (BioXCell, West Lebanon, NH) at 10 μg/mL and intercellular adhesion molecule 1 (ICAM-1) at 1 μg/mL. Briefly, 5 × 10^5^ cells were added to chambers coated with OKT3 and ICAM-1 for 5, 15, or 30 minutes at 37°C. Then the cells were fixed with 2% paraformaldehyde in PHEM buffer, permeabilized with 0.1% Triton-X100 for 5 minutes at room temperature, blocked with casein, and incubated with primary antibodies for 1 hour at room temperature in 2% donkey serum. Finally, cells were incubated with secondary antibodies (1:250) and mounted in antifade reagent (SlowFade Gold, Invitrogen). Control samples of secondary antibodies alone were included in each experiment. Imaging was performed by using a Zeiss LSM 710 confocal laser-scanning microscope with a ×63 1.4NA objective to study time-dependent recruitment of proteins. Image analysis and 3-dimensional reconstruction was performed with ImageJ software (http://imagej.en.softonic.com).

### Total internal reflection fluorescence microscopy

A Nikon Ti microscope was used for all total internal reflection fluorescence microscopy experiments.^27^ The instrument was fitted with a ×100 TIRF objective: NA, 1.49; Andor iXon EMCCD camera, 405-, 491-, 561-, and 633-nm laser lines; appropriate CFP/fluorescein isothiocyanate (FITC)/Cy3/Cy5 emission filters; SRIC cube (IRM); a programmable mechanized stage; and infrared autofocus (Perfect Focus, Nikon, Melville, NY). Cultured T cells were incubated in OK-DMEM medium without IL-2 for 2 hours, washed, and resuspended at a density of 10^7^ cells/mL in Hepes buffered saline containing 1% human serum albumin, PH 7.2 equilibrated to 37°C for injection into flow chambers.

### Statistics

Mann-Whitney *U* tests were applied for testing differences between groups (GraphPad Prism 5 for MacOS X; GraphPad Software, San Diego, Calif).

## Results

### Peripheral blood lymphopenia, lymphoid hyperplasia, and autoimmunity in patients with PHTS

**Fig E1:**
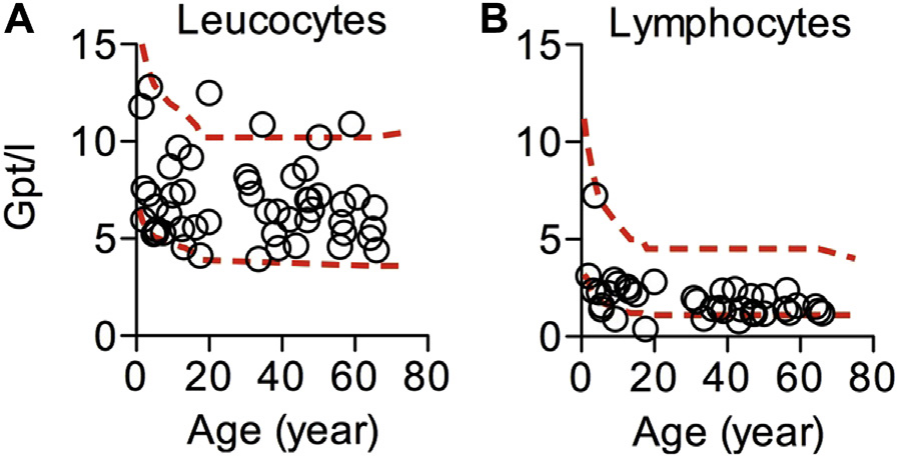
Leukocyte and lymphocyte counts in peripheral blood of patients with PHTS, with age indicated. **A,** Leukocyte counts. **B,** Lymphocyte counts. *Lines* represent the upper and lower normal reference.

In a group of 79 patients with pathogenic germline heterozygous mutations in *PTEN* (Fig 1, *A*), 43% present with autoimmunity, lymphoid hyperplasia, or both (autoimmunity in 27% and lymphoid hyperplasia in 24% of patients; Fig 1, *B*, and see Table E1). Susceptibility to infection was not detected (data not shown). Peripheral total blood leukocyte counts were normal in patients with PHTS. However, we observed in all age groups a significant reduction in peripheral lymphocyte numbers compared with those in control subjects (Fig 1, *C*, and see Fig E1 in this article's Online Repository at www.jacionline.org).

**Fig E2:**
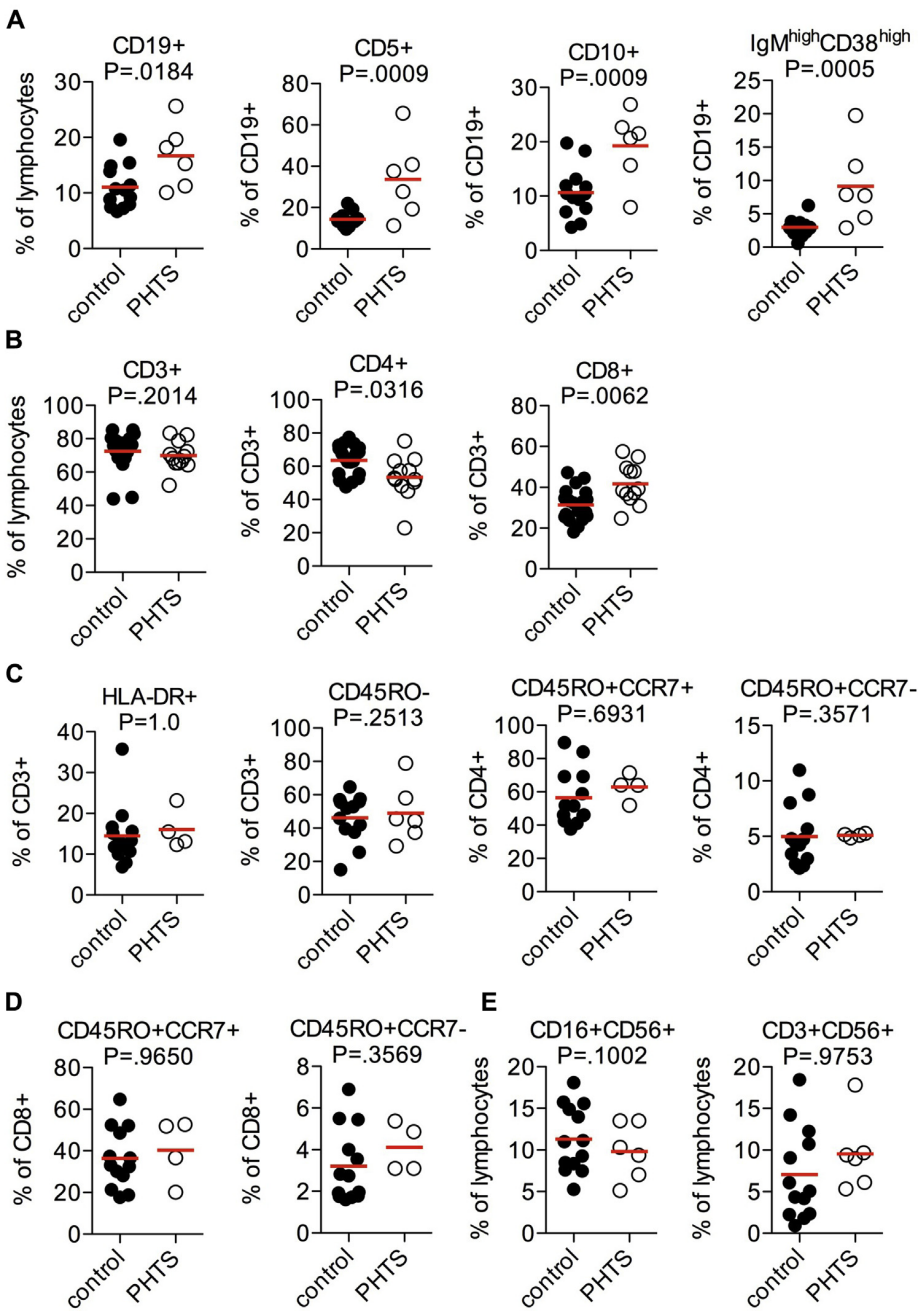
Lymphocyte subsets of patients with PHTS (proportions). **A,** Frequencies of CD19^+^, CD5^+^, CD10^+^ immature, and IgM^high^CD38^high^ transitional B cells. **B,** Frequencies of CD3^+^ T cells and percentages of CD4^+^ and CD8^+^ T cells among CD3^+^ T cells. **C,** Frequencies of HLA-DR^+^ activated T cells and CD45RO^−^ naive T cells, CD45RO^+^CCR7^+^ central memory CD4^+^ T cells, and CD45RO^+^CCR7^−^ effector memory CD4^+^ T cells. **D,** Frequencies of CD45RO^+^CCR7^+^ central memory CD8^+^ T cells and CD45RO^+^CCR7^-^ effector memory CD8^+^ T cells. **E,** Frequencies of CD16^+^CD56^+^ NK cells and CD3^+^CD56^+^ NKT cells. Each *dot* represents 1 patient. The mean is presented by the *red line*. Statistical differences were analyzed by using the Mann-Whitney test.

**Fig E3:**
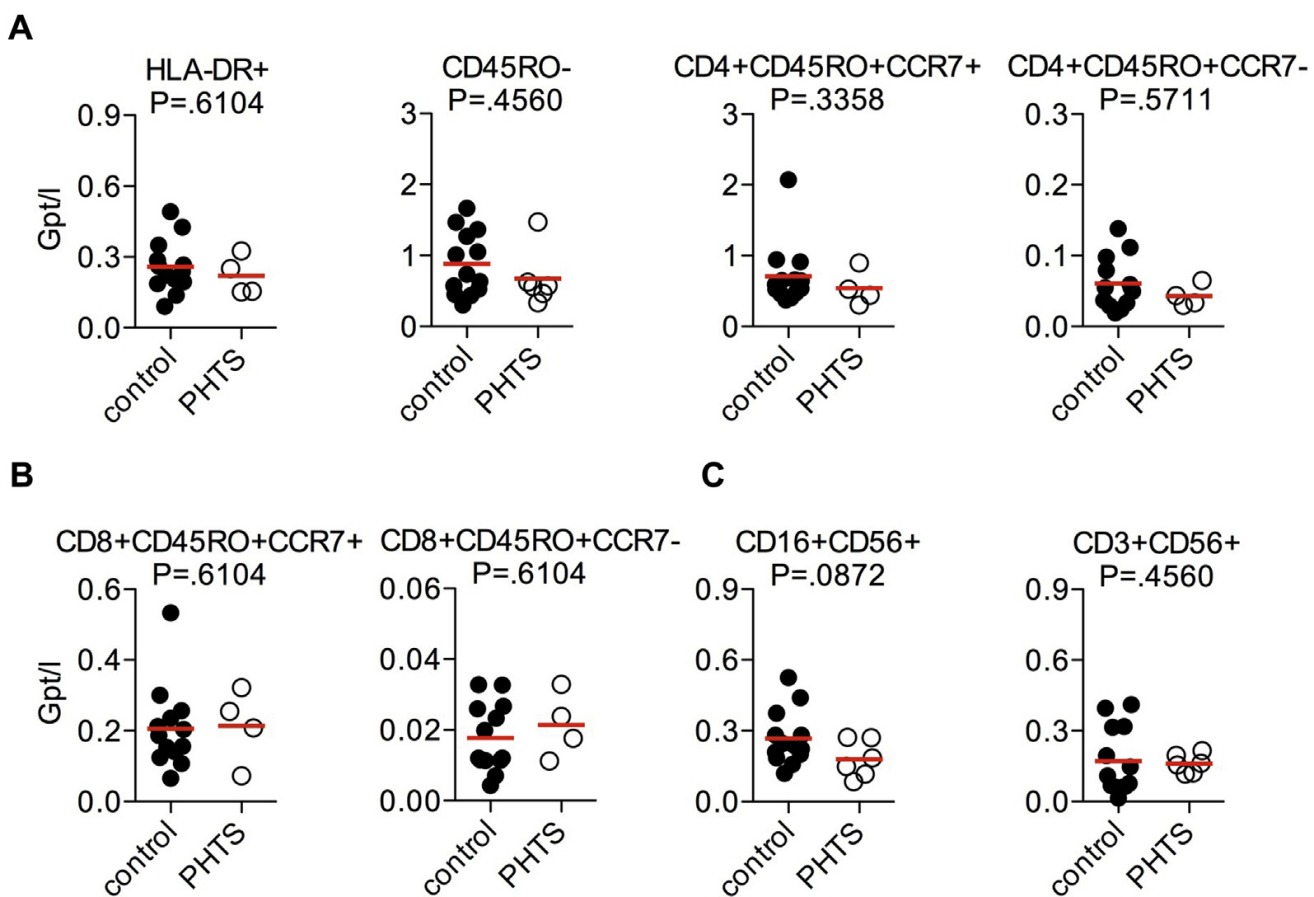
FACS analysis of T-cell subsets, NK cells, and NKT cells in peripheral blood of patients with PHTS (absolute numbers). **A,** Levels of HLA-DR^+^ activated T cells and CD45RO^−^ naive T cells, CD45RO^+^CCR7^+^ central memory CD4^+^ T cells, and CD45RO^+^CCR7^−^ effector memory CD4^+^ T cells. **B,** Levels of CD45RO^+^CCR7^+^ central memory CD8^+^ T cells and CD45RO^+^CCR7^−^ effector memory CD8^+^ T cells. **C,** Levels of CD16^+^CD56^+^ NK cells and CD3^+^CD56^+^ NKT cells. Each *dot* represents 1 patient. The mean is presented by the *red line*. Statistical differences were analyzed by using the Mann-Whitney test.

**Fig E4:**
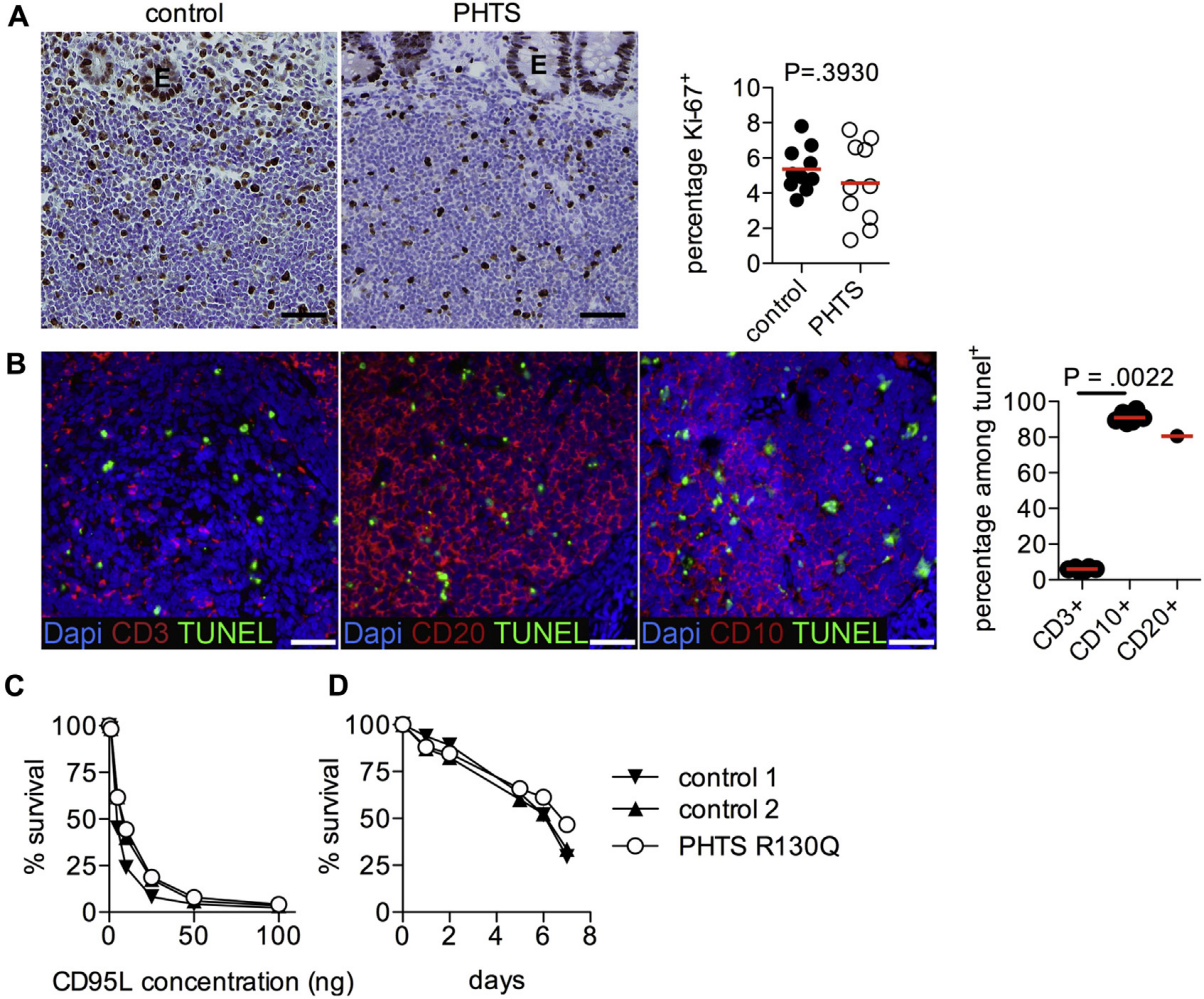
Proliferation and apoptosis in lymphocytes from patients with PHTS. **A,** Gut tissue sections from patients with PHTS or control subjects were stained for Ki-67. Cell proliferation was analyzed within the T-cell areas. *E*, Epithelium. **B,** CD3, CD20, and CD10 costaining with terminal deoxynucleotidyl transferase dUTP nick end-labeling *(TUNEL)* for apoptosis detection in control appendix sections. The graph demonstrates the percentage of TUNEL^+^ cells that are CD3^+^, CD20^+^, or CD10^+^. Differences were analyzed by using the Mann-Whitney test. *Scale bars* = 50 μm. *Symbols* represent individual patients. **C** and **D,***In vitro* apoptosis assays. Apoptosis was induced in PHA-activated T-cell blasts by means of CD95L (FasL) stimulation (Fig E4, *C*) or IL-2 deprivation (Fig E4, *D*). Percentages of surviving cells are shown for 1 patient with PHTS (p.R130Q) and 2 healthy control subjects. Similar results have been obtained by using analysis of 6 further patients with PHTS with different mutations in an independent experiment (data not shown). *Dapi*, 4′, 6′-Diamidino-2-phenylindole.

We then characterized blood lymphocyte subsets using FACS (Fig 1, *D* and *E*, and see Fig E2 in this article's Online Repository at www.jacionline.org). There was a significant accumulation of CD5^+^, CD10^+^, and IgM^high^CD38^high^ transitional B cells (Fig 1, *D*) and a significant reduction in CD4^+^ cell numbers, resulting in an alteration in the CD4^+^/CD8^+^ cell ratio (Fig 1, *E*, and see Fig E2). Patients with PHTS have largely normal naive, central, and effector memory T (Tmem) cell numbers, as well as natural killer (NK) or NKT-cell numbers (see Figs E2, *C* and *D*, and E3 in this article's Online Repository at www.jacionline.org). In contrast to CD10^+^/CD20^+^ germinal center B cells,^11^ proliferation rates in the T-cell areas of mucosa-associated lymphoid tissue (MALT) were similar between patients with PHTS and control subjects, and the apoptosis rate of CD3^+^ T lymphocytes *in situ* was comparatively low (see Fig E4, *A* and *B*, in this article's Online Repository at www.jacionline.org). To assess apoptosis of T cells from patients with PHTS *in vitro*, we investigated FAS ligand (CD95L)–induced or IL-2 deprivation–induced apoptosis of T-cell blasts (see Fig E4, *C* and *D*). No significant changes in the apoptosis rate of T-cell blasts were detected. These findings suggest that heterozygous pathogenic PTEN mutations have substantial effects on B-cell differentiation and CD4^+^ T cell numbers, but surprisingly little effect on overall T-cell development, proliferation, and apoptosis.

### Normal frequency but increased proliferation of FOXP3^+^ T cells *in situ*

**Fig 2:**
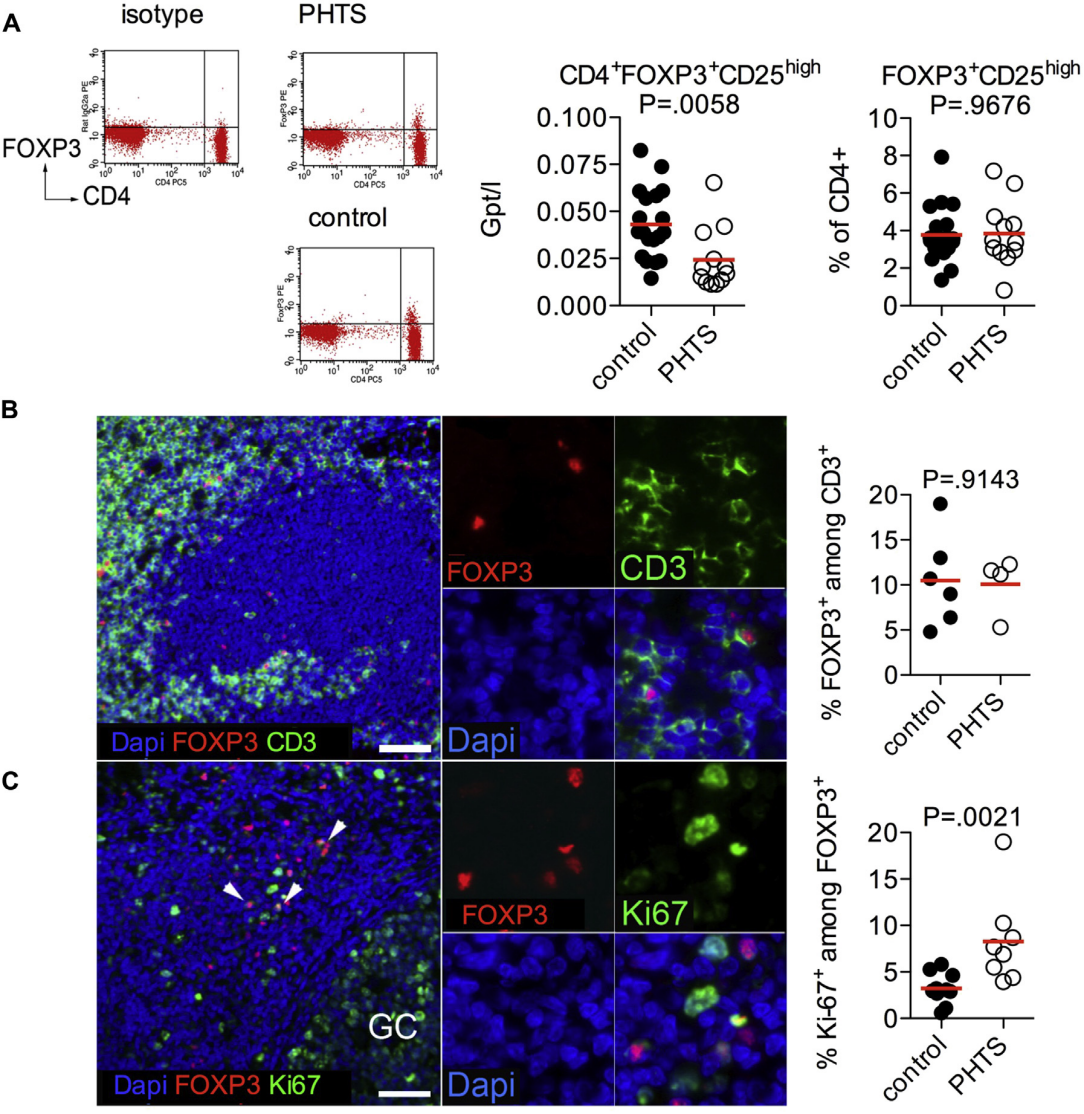
Treg cells in patients with PHTS. **A,** Absolute counts and frequencies of Treg cells in blood from patients with PHTS or control subjects. **B,** Percentages of FOXP3^+^ cells among CD3^+^ cells were analyzed in MALT from control subjects and patients. Representative staining of tissue sections from patients with PHTS is shown. **C,** FOXP3 and Ki-67 expression in MALT. Percentages of Ki-67^+^ proliferating FOXP3^+^ Treg cells were analyzed in control and patient samples. *Arrows* mark Ki-67^+^FOXP3^+^ Treg cells. *Scale bars* in all images represent 50 μm. *Dapi*, 4′, 6′-Diamidino-2-phenylindole; *GC*, location of a germinal center.

Increased PI3K signaling has been shown to suppress Treg cell differentiation and function,^14^, ^17^, ^28^ in part through inhibitory effects on Foxo1 and Foxo3a.^19^, ^21^, ^22^ Therefore we investigated the frequency of FOXP3^+^ Treg cells, as well as FOXP3 expression levels, in patients with defects in *PTEN*. Although absolute Treg cell numbers are lower in patients with PHTS, because of the CD4 lymphopenia, similar frequencies of Treg cells were found in peripheral blood, as well as in MALT, in patients with PHTS compared with control subjects (Fig 2, *A* and *B*). Similar to studies in mice,^4^ frequencies of Ki-67^+^FOXP3^+^ proliferating cells within the lymphoid tissues of patients with PHTS were increased (Fig 2, *C*).

**Fig 3:**
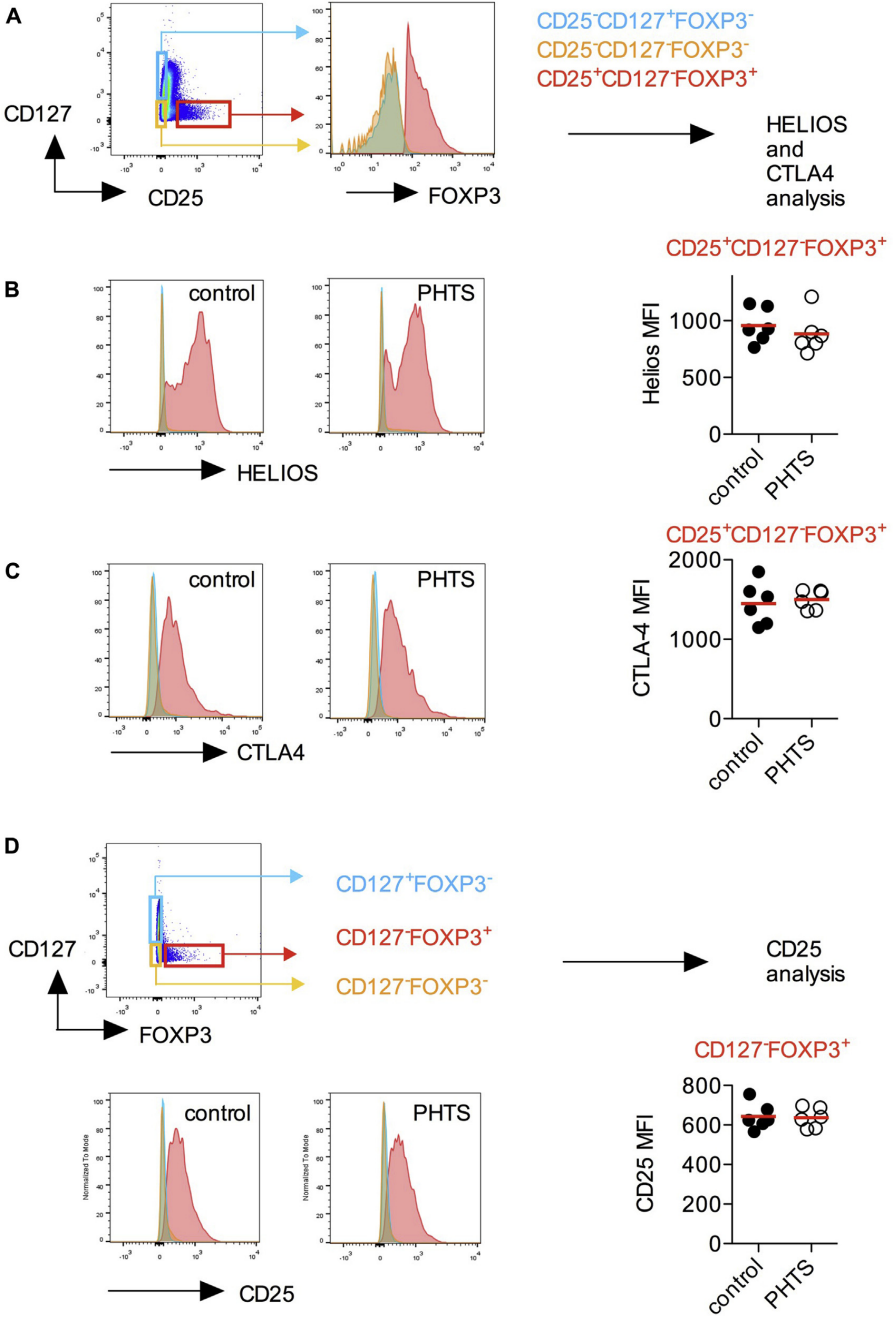
Phenotype of Treg cells in patients with PHTS. **A,** CD3^+^CD4^+^FOXP3^+^CD25^+^CD127^−^ cells (Treg cells) were compared with FOXP3^−^CD25^−^CD127^+^ (naive and central Tmem cells) and FOXP3^−^CD25^−^CD127^−^ cells (effector T cells). **B,** Expression levels of Helios are shown as a histogram and quantified in Treg cells. **C,** Expression levels of CTLA-4 are shown as a histogram and quantified in Treg cells. **D,** CD3^+^CD4^+^FOXP3^+^CD127^−^ (Treg) cells were compared with FOXP3^−^CD127^+^ (naive and central Tmem) and FOXP3^−^CD127^−^ (effector T cells) cells. CD25 expression in CD4^+^FOXP3^+^ Treg cells is shown as a histogram and quantified in Treg cells. No statistical differences were detected by using the Mann-Whitney *U* test.

We next measured markers of Treg cell function and suppressive activity. We show that FOXP3^+^ Treg cells of patients with PHTS express normal levels of Helios and CTLA-4 (Fig 3, *A-C*). Because murine FOXP3^cre^Pten^fl/fl^ Treg cells show decreased CD25 expression,^5^ we compared CD25 expression of FOXP3^+^ Treg cells and found that human Treg cells with heterozygous loss of PTEN remain phenotypically normal (Fig 3, *D*).

Taken together, these data suggest that PTEN deficiency reduces FOXP3^+^ Treg cell blood numbers and affects their proliferation *in situ* but has no effect on their phenotype.

### Negative control of PI3K activity downstream of TCR activation in Treg cells

**Fig 4:**
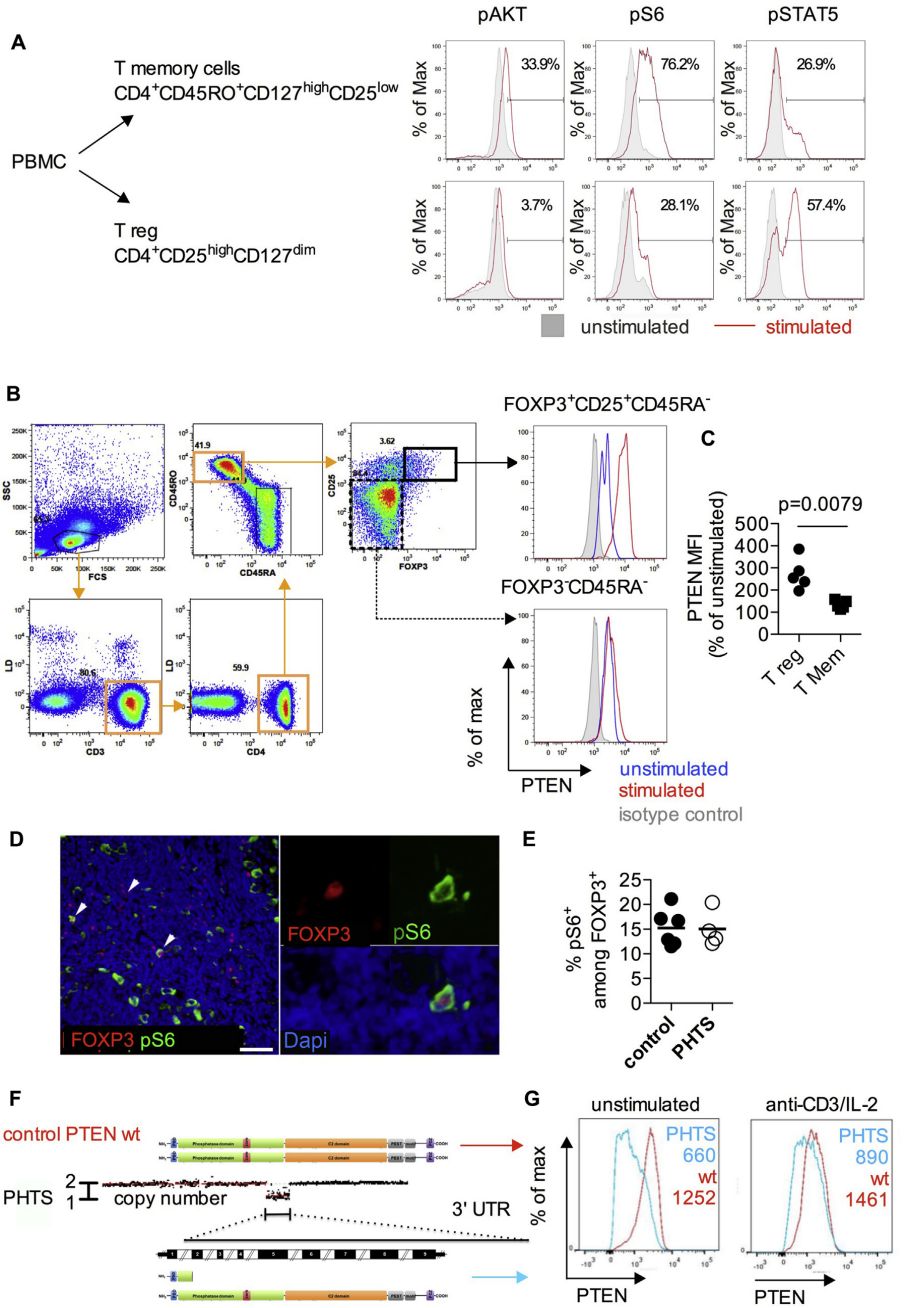
PTEN activity and PI3K signaling in natural Treg cells. **A,** FACS-sorted Treg cells (CD4^+^CD25^high^CD127^low^) and Tmem cells (CD4^+^CD45RO^+^CD127^high^CD25^low^) were left unstimulated or subjected to anti-CD3/CD28 and IL-2 stimulation for 10 minutes. Cellular levels of pAKT, pS6, and phosphorylated signal transducer and activator of transcription 5 *(pSTAT5)* in each cell type with or without stimulation were analyzed by using Phosflow. **B** and **C,** PBMCs from healthy donors were rested or stimulated for 24 hours with anti-CD3 and anti-CD28 T-cell activator beads in the presence of IL-2. PTEN expression in FOXP3^−^ nonregulatory and FOXP3^+^ Treg cell populations among total PBMCs was measured by using FACS to detect changes in PTEN protein levels after TCR and IL-2 receptor stimulation. **D,** MALT sections were stained for FOXP3^+^ cells, as well as phosphorylation of the S6 ribosomal protein (p[Ser235/236]S6). *Scale bar* = 50 μm. **E,** Percentage of pS6^+^ expressing cells among FOXP3^+^ cells in patients with PHTS versus control subjects determined by using immunohistochemical staining. *Symbols* represent individual patients, and *lines* represent means. Differences were analyzed by using the Mann-Whitney *U* test. **F,** Multiplex ligation-dependent probe amplification copy number analysis revealing a microdeletion spanning from exon 2 to exon 9 and a 3′ untranslated region *(UTR)* of PTEN. Protein representation of a wild-type *(wt)* subject and a patient with PHTS with the microdeletion (del E2-9) are shown for comparison. **G,** PTEN expression in CD3^+^ T cells from a control donor or a patient with PHTS (del E2-9, PTEN microdeletion) was determined by using FACS with an mAb that recognizes the C-terminal region of PTEN, allowing selective detection of the nonmutated copy of PTEN protein. PTEN levels were measured in cells with or without anti-CD3/CD28 and IL-2 stimulation for 24 hours.

We next investigated TCR- and IL-2–induced activation of the PI3K/AKT pathway in FACS-sorted Treg cells and CD4^+^ Tmem cells from healthy donors using Phosflow analysis. We found lower levels of pAKT and pS6 on TCR/IL-2 stimulation in Treg cells compared with Tmem cells, which is consistent with the importance of sustained negative regulation of PI3K signaling for Treg cell function (Fig 4, *A*). By contrast, Treg cells had heightened signal transducer and activator of transcription 5 phosphorylation in IL-2–stimulated Treg cells compared with Tmem cells, which is consistent with higher levels of CD25 expression in Treg cells.

All CD4^+^ T-cell populations, including naive and Tmem cells, as well as Treg cells, expressed PTEN under steady-state conditions. After TCR and IL-2 stimulation, Treg cells had substantially upregulated PTEN levels, whereas FOXP3^−^ T cells did not (Fig 4, *B* and *C*).

To investigate PTEN/AKT/mTOR complex 1 (mTORC1) signaling in patients with PHTS, we analyzed the frequency of FOXP3^+^ cells with S6 ribosomal protein phosphorylation *in situ*. There was a similar frequency of S6-phosphorylated FOXP3^+^ cells in control subjects and patients with PHTS (Fig 4, *D* and *E*), indicating that despite the PTEN insufficiency/dysfunction, the signaling pathway downstream of mTORC1 is not hyperactivated.

It is possible that heterozygous loss of PTEN could be compensated by increased expression of the functional allele. We examined PTEN levels in CD3^+^ cells from a patient with a heterozygous *PTEN* microdeletion, allowing the mutated and wild-type copies to be distinguished by a C-terminal anti-PTEN antibody (Fig 4, *F*). Heterozygous loss of PTEN resulted in approximately half the cellular level of PTEN protein (Fig 4, *G*).

Collectively, these observations support the paradigm that PI3K activity in Treg cells is tightly controlled, but surprisingly, heterozygous loss of PTEN does not increase PI3K signaling downstream of PTEN nor does it selectively reduce FOXP3^+^ T-cell numbers or impair their phenotype.

### Functional significance of PHLPP in Treg cells

**Fig E5:**
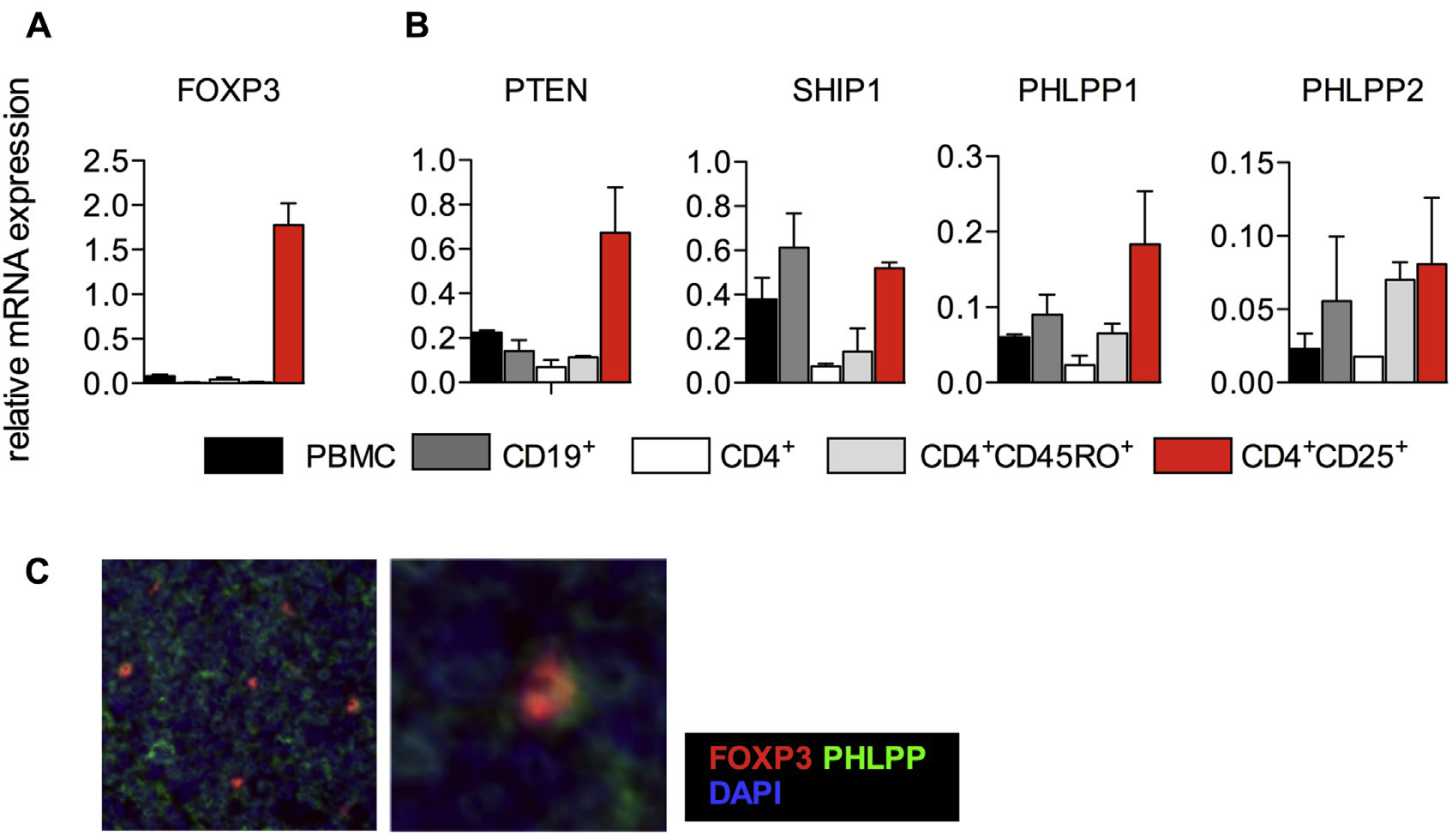
Phosphatases in PBMCs and blood-derived lymphocyte subsets. **A** and **B,** mRNA gene expression analysis in PBMCs compared with FACS-sorted B cells, whole CD4^+^ T cells, Tmem cells, and CD4^+^CD25^+/high^ Treg cells. Comparative gene expression analysis of FOXP3 (Fig E5, *A*) and different phosphatases (PTEN, SHIP1, PHLPP1, and PHLPP2; Fig E5, *B*) regulating the PI3K/AKT/mTOR signaling pathway. Values are normalized to 18S rRNA. Means and SDs are shown for triplicate values. **C,** Immunohistochemical detection of PHLPP protein in FOXP3^+^ cells. Images show representative PHLPP staining in FOXP3^+^ cells in a control appendix section. *DAPI*, 4′, 6′-Diamidino-2-phenylindole.

We hypothesized that the insufficiency in PTEN might be functionally compensated by other phosphatases. We performed gene expression analysis of phosphatases targeting phosphatidylinositol (3,4,5)-trisphosphate or pAKT in Treg cells compared with PBMCs, CD4^+^ T cells, and B cells. Indeed, not only PTEN but also PHLPP1, Src homology domain 2–containing inositol phosphatase (SHIP) 1, and PHLPP2 were expressed at relatively high levels in FOXP3-expressing cells, and PHLPP expression in FOXP3^+^ cells was confirmed by means of immunohistochemistry (see Fig E5 in this article's Online Repository at www.jacionline.org).

**Fig E6:**
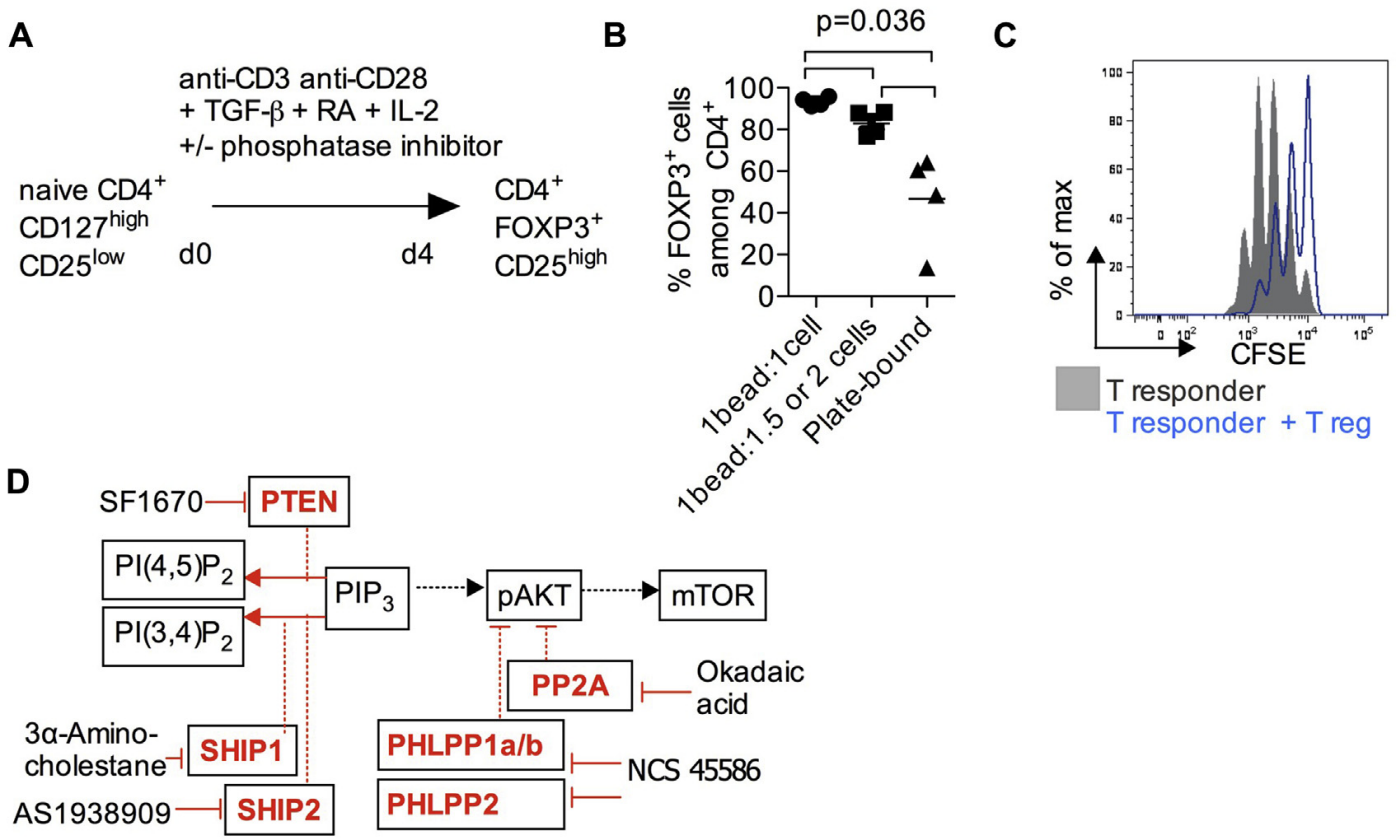
iTreg assay and blockade of iTreg generation by phosphatase-specific inhibitors. **A,***In vitro* differentiation of naive T cells into FOXP3 iTreg cells. **B,** Effects of TCR signal strength on FOXP3 induction. TCR stimulation was provided by T-cell activator beads or plate-bound anti-CD3 (10 μg/mL)/soluble anti-CD28 (1 μg/mL). **C,***In vitro* T-cell proliferation suppression assay. Carboxyfluorescein succinimidyl ester *(CFSE)*–labeled naive (responder) T cells were cocultured with anti-CD3/anti-CD28 beads in the presence and absence of iTreg cells. Responder T-cell proliferation was measured by means of CFSE dilution. Data are representative of 2 independent experiments. **D,** Small-molecule inhibitors were used to block various phosphatases (PTEN, PHLPP1/2, SHIP1, SHIP2, and PP2A) that provide negative regulation at different points of the PI3K/AKT/mTOR signaling pathway.

**Fig E7:**
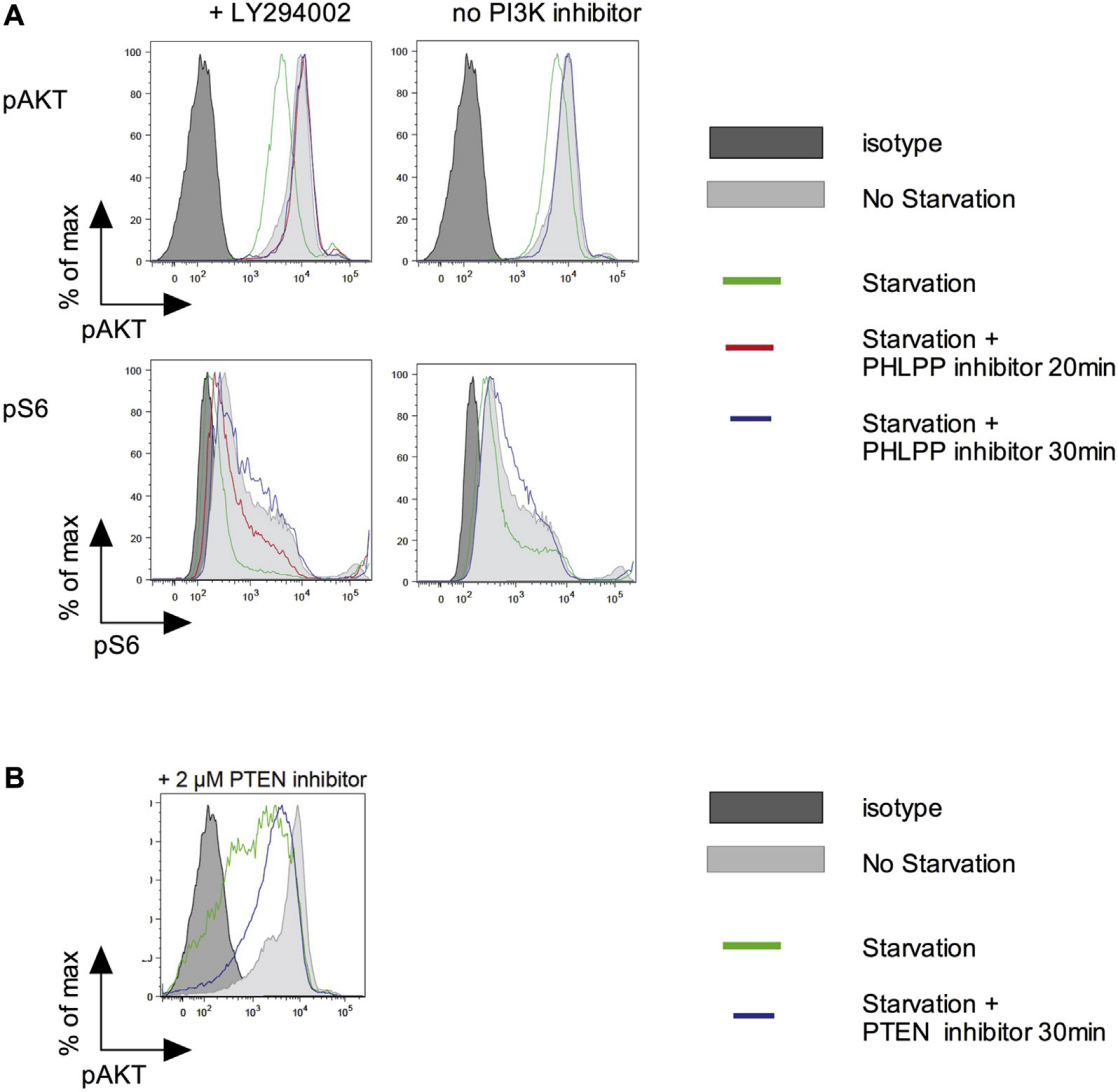
Effects of the small-molecule inhibitors of PHLPP and PTEN on AKT/S6 phosphorylation. **A,** Levels of pAKT and pS6 in HEK293T cells with or without PHLPP inhibitor treatment. HEK293T cells were plated for 24 hours in Dulbecco modified Eagle medium supplemented with 10% FCS before serum starvation. Cells were then incubated for 24 hours in the presence or absence of 10% FCS (starvation or no starvation, respectively). Where appropriate, the PI3K inhibitor LY294002 was added to cells 6 hours after the start of serum starvation and left on for 18 hours to further reduce baseline levels of pAKT in serum-starved cells. Before fixation of cells for Phosflow, cells were exposed to the PHLPP inhibitor for 20 or 30 minutes. pAKT and pS6 levels were measured by using Phosflow. Data are representative of 2 independent experiments. **B,** Levels of pAKT in HEK293T cells with or without PTEN inhibitor treatment. HEK293T cells were plated for 24 hours in Dulbecco modified Eagle medium supplemented with 10% FCS before serum starvation. Cells were then incubated for 24 hours in the presence or absence of 10% FCS (starvation or no starvation, respectively). Before fixation of cells for Phosflow, cells were exposed to the PTEN inhibitor SF1670 for 30 minutes. pAKT levels were measured by using Phosflow.

**Fig 5:**
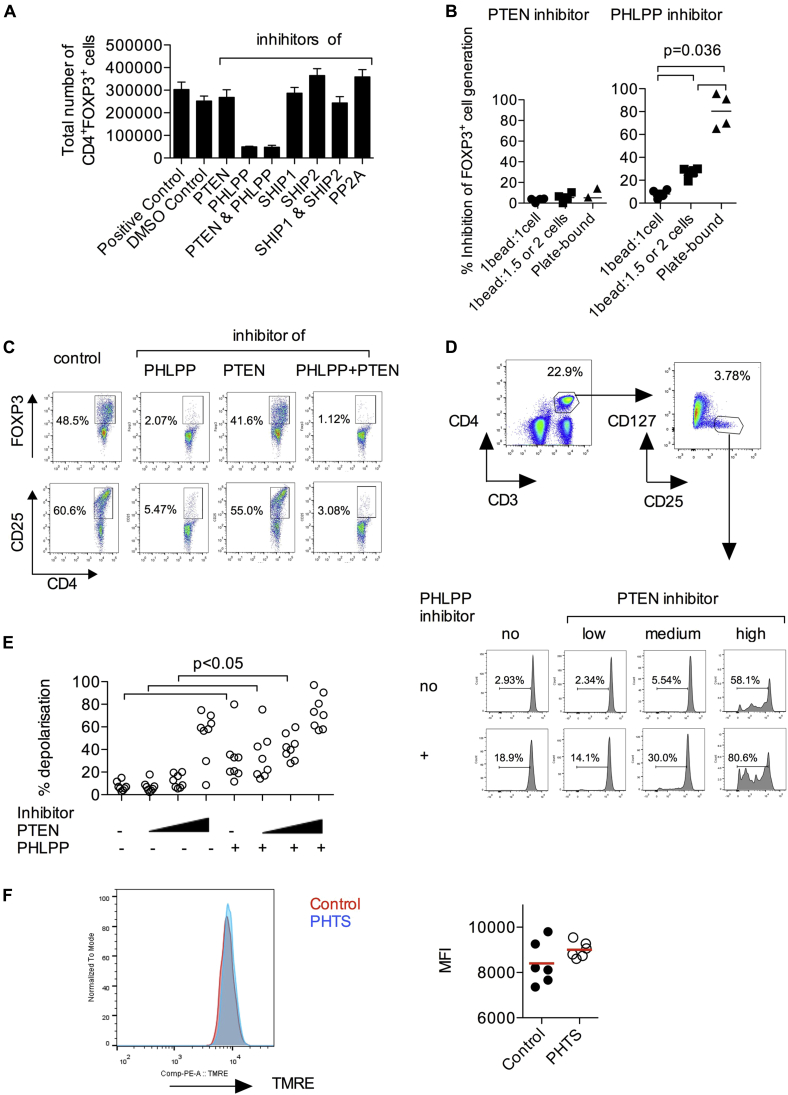
Functional complementation between PTEN and PHLPP in Treg cells. **A,** Effects of small-molecule inhibitors of PTEN, PHLPP, SHIP1, SHIP2, and PP2A on iTreg cell differentiation *in vitro*. CD4^+^ naive human T cells were cultured for 4 days in the presence of various inhibitors under iTreg cell–inducing conditions. A dimethyl sulfoxide concentration-matched control was used. Total numbers of CD4^+^FOXP3^+^ cells for each condition at the end of the 4-day culture are shown. *Bars* represent means ± SDs for a culture performed in quadruplicate. Results are representative of 3 independent experiments. iTreg cell induction was performed at medium TCR stimulation signal strength (1 bead: 2 cells). **B,** Effects of TCR stimulation signal strength on the extent of blockade of iTreg cell generation by inhibitors of PTEN or PHLPP. **C,** Representative FACS plots demonstrating the effects of the PTEN and PHLPP inhibitors on iTreg cell differentiation. **D** and **E,** Mitochondrial depolarization in blood Treg cells after PTEN and/or PHLPP inhibition. Mitochondrial membrane potential was measured by using the TMRE assay in CD4^+^CD25^high^CD127^low^ Treg cells treated with a PTEN inhibitor at 0.4, 2 (IC_50_), or 4 μmol/L alone or in combination with a PHLPP inhibitor (used at an IC_50_ of 70 μmol/L). Dimethyl sulfoxide concentrations are equal in all conditions. Data are pooled from 8 healthy donors. Each *point* represents 1 donor, and differences have been assessed by using the Mann-Whitney *U* test. **F,** Mitochondrial depolarization in blood Treg cells from patients with PHTS and control subjects. TMRE fluorescence in Treg cells is shown as a histogram and quantified.

To interrogate the functional role of those phosphatases, we performed an *in vitro* induced regulatory T (iTreg) cell inducer assay by using increasing numbers of T-cell activator beads (see Fig E6, *A* and *B*, in this article's Online Repository at www.jacionline.org). The strongest induction of FOXP3^+^ iTreg cells that suppressed proliferation of naive CD4^+^ T cells *in vitro* was achieved by using a high ratio of beads to cells (see Fig E6, *B* and *C*). We investigated the effects of various small-molecule inhibitors against phosphatases regulating the PI3K pathway (see Fig E6, *D*). The induction of iTreg cells was significantly reduced by inhibition of PHLPP1/2 and simultaneous inhibition of PHLPP1/2 and PTEN but not by PTEN inhibition alone or by inhibition of SHIP1, SHIP2, or protein phosphatase 2A (PP2A; Fig 5, *A-C*). Therefore PHLPP plays a key role in the conversion of naive CD4^+^ T cells to iTreg cells. We confirmed the target activities of the PTEN and PHLPP inhibitors by measuring pAKT and pS6 levels in HEK293T cells (see Fig E7 in this article's Online Repository at www.jacionline.org). The effects of PHLPP inhibition on iTreg cell generation was highest with the plate-bound TCR stimulus (Fig 5, *B*). Collectively, our data suggest that PHLPP is expressed in Treg cells *in situ* and *ex vivo* and that PHLPP activity is essential for the induction of iTreg cells.

**Fig E8:**
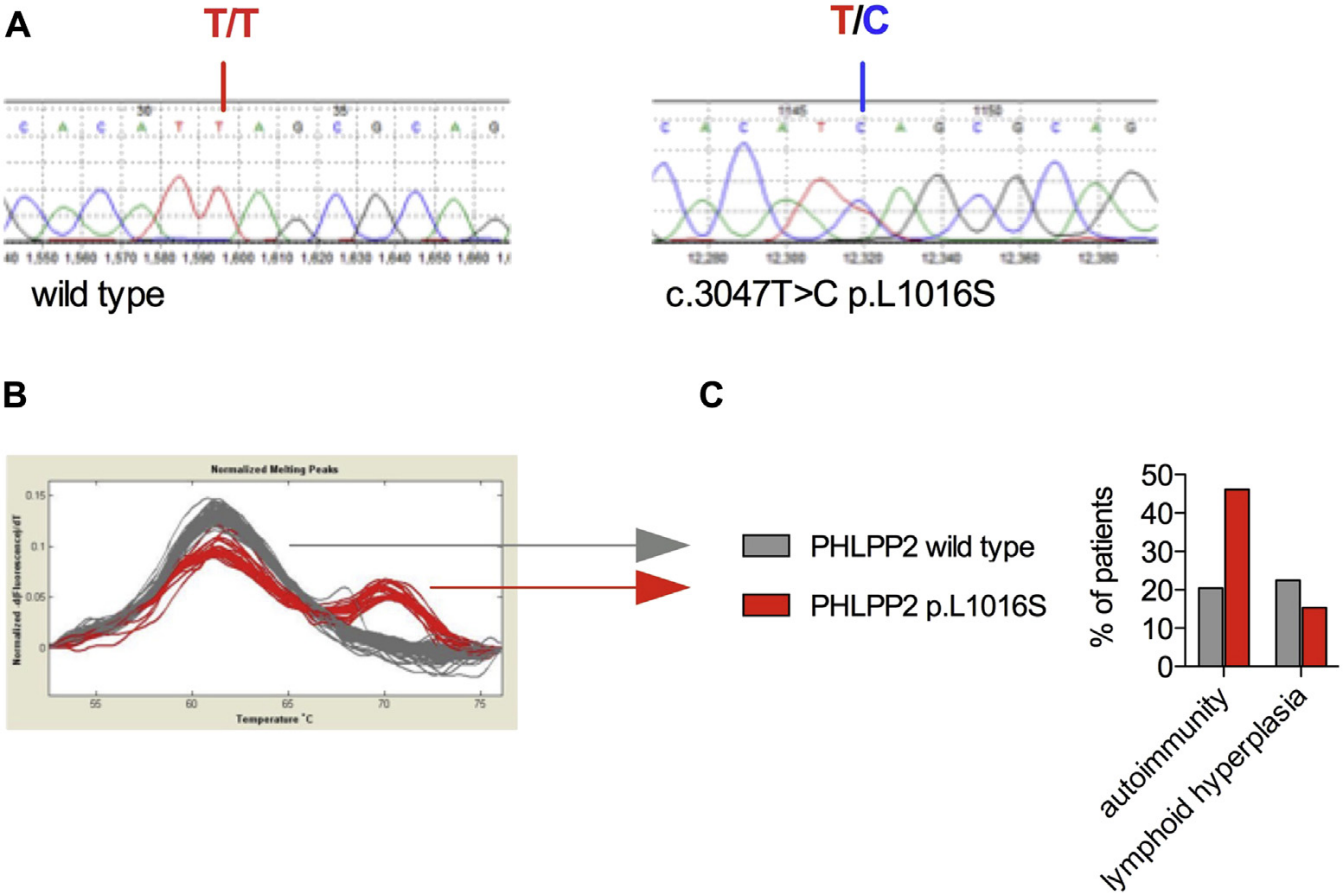
Effect of PHLPP2 polymorphism p.L1016S in patients with pathogenic PTEN mutations. **A,** Sanger sequencing of wild-type and c.3047T>C (p.L1016S) variants. **B,** LightScanner analysis. **C,** PHLPP2 genotype-phenotype analysis. Sixty-six patients with PHTS were analyzed for the L1016S variant and autoimmune and lymphoid hyperplasia phenotype. Potential gene-gene interaction between PTEN and PHLPP2 was investigated by determining whether the hypomorphic polymorphism in PHLPP2 (p.L1016S) with reduced dephosphorylation activity at the C-terminal hydrophobic motif Ser473 of AKT^E9^ has a detectable effect on the immune phenotype in patients with PHTS. The p.L1016S variant is present in about one fifth of the human population^E9^ and was similarly frequently detected in the PHTS cohort. Although there is no literature to suggest that this polymorphism has major relevance for the development of autoimmunity in the general population with wild-type PTEN, we hypothesized that this polymorphism could be relevant for patients who harbor a pathogenic PTEN mutation and would therefore likely have a different PI3K-AKT signal activation threshold. There was a nonsignificant trend toward an increased frequency of autoimmunity and no significant difference in lymphoid hyperplasia between patients with PHTS with wild-type or L1016S variants. Our data suggest that heterozygous loss of PHLPP2 phosphatase activity has a potential effect on autoimmunity in those patients, but despite analysis of 66 patients with PHTS, this is underpowered.

Given the importance of PI3K/mTOR in mitochondrial metabolism, we next examined the decrease in mitochondrial membrane potential in blood-derived Treg cells treated with increasing concentrations of the PTEN inhibitor either alone or in combination with the PHLPP inhibitor (Fig 5, *D* and *E*). We found that simultaneous blockade of both phosphatases resulted in significantly decreased membrane potential compared with blocking PHLPP or PTEN alone. Similar to intermediate PTEN inhibitor concentrations, the TMRE assay performed on patients' Treg cells with haploinsufficient PTEN showed normal mitochondrial membrane potential (Fig 5, *F*), suggesting functional compensation for PTEN. Further studies are needed to clarify whether the loss-of-function PHLPP2 polymorphism p.L1016S is associated with autoimmunity in patients with PHTS (see Fig E8 in this article's Online Repository at www.jacionline.org).

### PTEN, PHLPP, and NHERF1 in Treg cells

**Fig E9:**
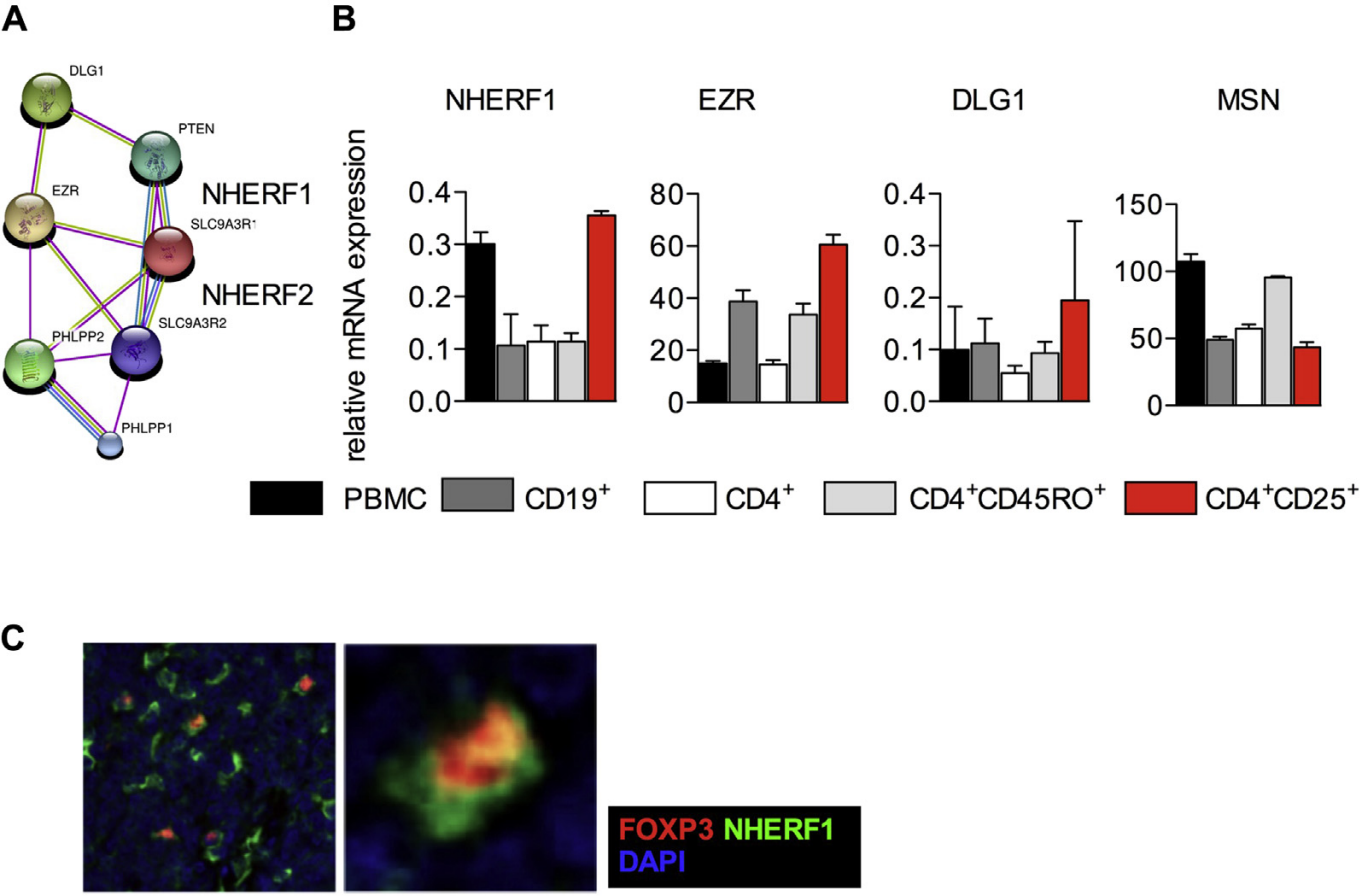
Expression of scaffold proteins in Treg cells. **A,** Molecular interactions between PTEN and PHLPP1/2 suggest a protein network that links those phosphatases with the structural scaffold proteins NHERF1/2 and the membrane linker ezrin (*EZR*; String database 9.0, high-confidence setting). **B,** mRNA gene expression analysis in PBMCs compared with FACS-sorted B cells, whole CD4^+^ T cells, Tmem cells, and CD4^+^CD25^+/high^ Treg cells. Comparative gene expression analysis of the structural proteins NHERF1, EZR, DLG1, and moesin *(MSN)* is shown. Values are normalized to the 18S rRNA level in each cell population. Means and SDs are shown for triplicate values. NHERF2 expression was not detected (data not shown). **C,** Immunohistochemical detection of NHERF1 expression in FOXP3^+^ cells. Images show representative NHERF1 staining in the control appendix section. *DAPI*, 4′, 6′-Diamidino-2-phenylindole.

We next investigated interaction partners of PTEN and PHLPP using the STRING database (http://string-db.org). This indicated that the phosphatases PTEN and PHLPP can form shared protein-protein interaction networks through scaffold proteins, including NHERF1 (encoded by *SLC9A3R1*), NHERF2 (encoded by *SLC9A3R2*), and scaffold protein discs, large homolog 1 (DLG1; see Fig E9, *A*, in this article's Online Repository at www.jacionline.org).

To validate this *in silico* prediction, we performed mRNA gene expression analysis. Treg cells showed a 3-fold higher level of NHERF1 mRNA compared with PBMCs or whole CD4^+^ T cells, CD4^+^CD45RO^+^ Tmem cells, and B cells (see Fig E9, *B*). The membrane-cytoskeleton linkers ezrin and moesin and DLG1 all displayed relatively high expression in Treg cells. NHERF1 is differentially expressed among lymphocytes in the T-cell area of human appendix sections, and FOXP3^+^ cells express medium to high levels of NHERF1 protein *in situ* (see Fig E9, *C*).

**Fig E10:**
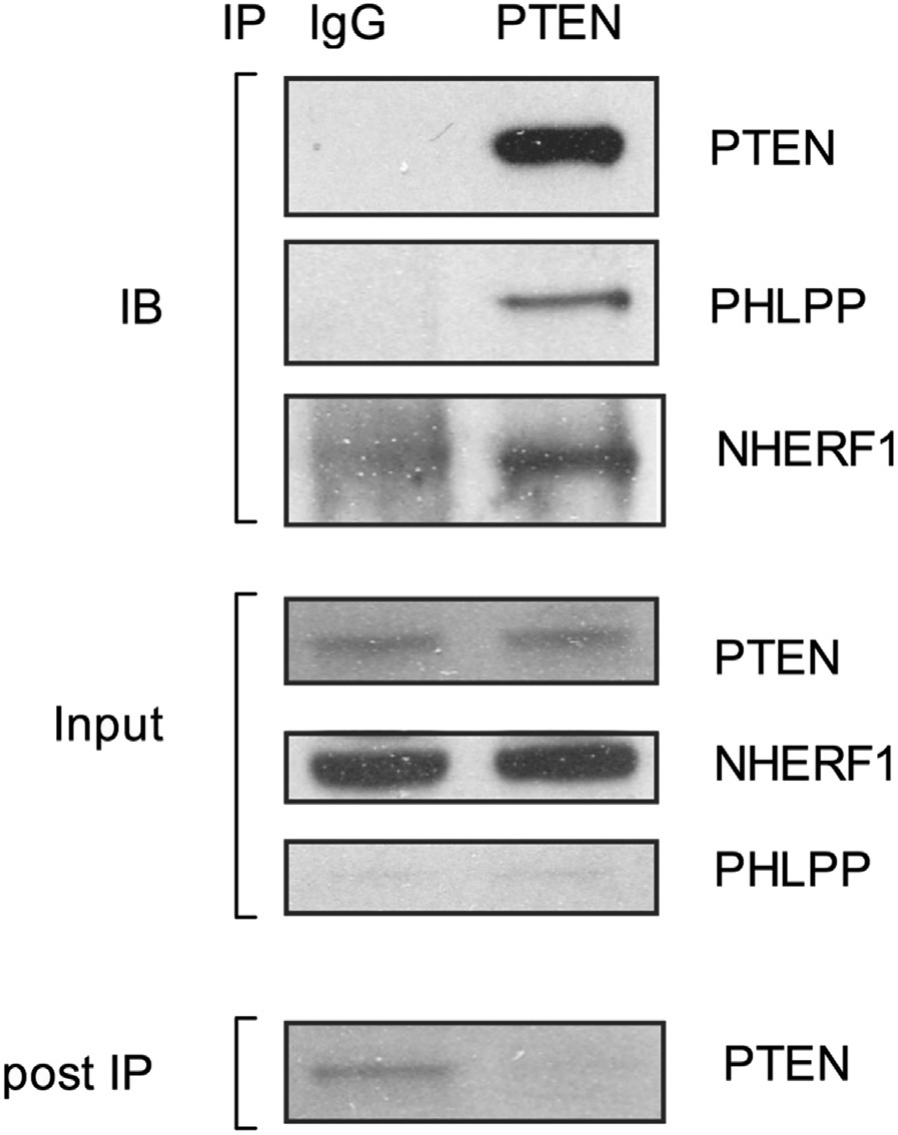
Interaction of PTEN with PHLPP and NHERF1 in NIH/3T3 cells. Immunoprecipitation of PTEN followed by Western blot analysis for PTEN, NHERF1 and PHLPP in NIH/3T3 cells. Enrichment of PHLPP and NHERF1 in the immunoprecipitated anti-PTEN fraction but not in control IgG precipitate *(IB)* demonstrates physical association between PTEN and NHERF1 or PHLPP. For comparison, PTEN, PHLPP, and NHERF1 levels are shown before *(Input)* and after immunoprecipitation (supernatant *post IP*).

NHERF1 is a candidate for mediating active assembly of PTEN and PHLPP. It contains PDZ domains that can link PTEN and PHLPP phosphatases through their PDZ-binding motifs.^29^, ^30^, ^31^ We confirmed physical association between NHERF1, PTEN, and PHLPP by means of immunoprecipitation of PTEN in the NIH/3T3 cell line, which expresses all 3 proteins constitutively (see Fig E10).

### Recruitment of PTEN, PHLPP, and NHERF1 to the immunologic synapse on TCR stimulation in Treg cells

**Fig 6:**
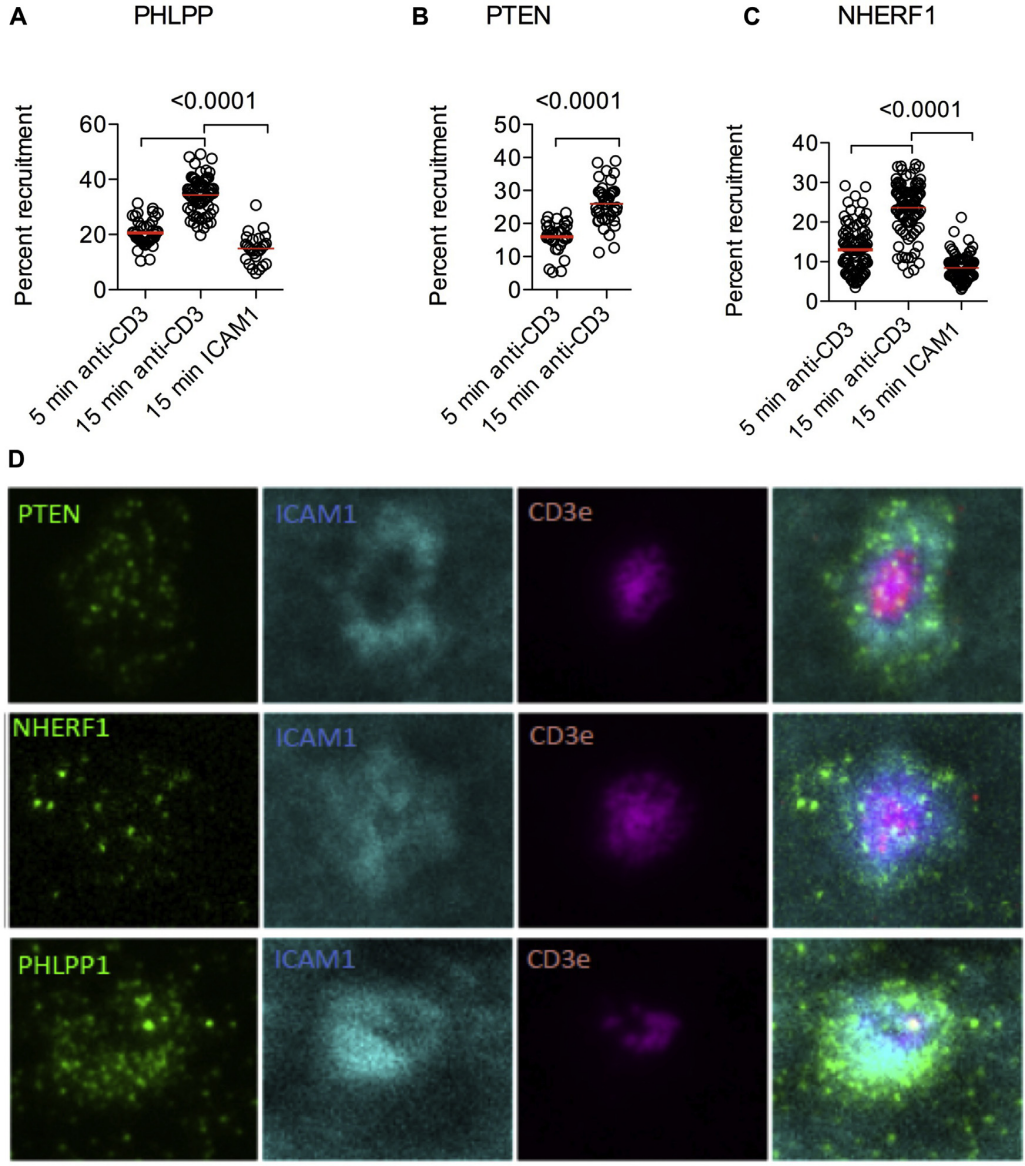
Phosphatase and NHERF1 assembly at the immunologic synapse. **A-C,** Laser-scanning microscopy of CD4^+^CD25^+^ T cells stimulated on CD3- and ICAM-1–coated glass slides. Time-dependent accumulation of PHLLP, PTEN, and NHERF1 after anti-CD3–mediated TCR activation was assessed by measuring protein accumulation in the planar layer adjacent to the glass slide. Exposure to ICAM-1 without TCR engagement served as a negative control. *Dots* represent individual cells analyzed. **D,** PTEN, NHERF1, and PHLLP subcellular localization in relation to the central supramolecular activation complex (indicated by CD3 staining) and peripheral supramolecular activation complex (indicated by ICAM-1 staining) was analyzed by using TIRF microscopy. Differences were analyzed by using the Mann-Whitney *U* test.

Through their association with membrane-cytoskeleton linkers, phosphatases can assemble into a network that allows rapid reconfiguration of their subcellular distribution on TCR stimulation. Association between phosphatases and scaffold components in Treg cells suggests a mechanism that allows polarized phosphatase activity at sites where regulated PI3K signaling is required. A likely place for such polarized phosphatase activity is the immunologic synapse. Therefore we quantified the polarization of PHLPP, PTEN, and NHERF1 using confocal laser-scanning microscopy. CD4^+^CD25^+^ Treg cells were activated by exposure to anti-CD3– and ICAM-1–coated glass slides. Within 15 minutes, PHLPP, PTEN, and NHERF1 accumulated toward the anti-CD3– and ICAM-1–coated glass surface but not toward the nonactivating ICAM-1–coated surface (Fig 6, *A-C*), suggesting that both phosphatases and NHERF1 actively accumulate at the site of the TCR ligation–triggered cellular signaling complex.

To confirm that these molecules are being recruited to the immune synapse, we used total internal reflection fluorescence microscopy on supported planar lipid bilayers. Consistent with the results from confocal imaging experiments, we observed clustering of PTEN, NHERF1, and PHLPP in the peripheral supramolecular activation complex within the immunologic synapse (Fig 6, *D*). Our observations suggest that a dynamic PTEN-NHERF1-PHLPP protein network is initiated after TCR stimulation in Treg cells, allowing simultaneous recruitment of the phosphatases to the site of TCR activation.

## Discussion

Because PTEN controls PI3K/AKT/mTOR signaling and patients with PHTS present with significant changes in the T- and B-cell compartment associated with autoimmunity, colitis, and lymphoid hyperplasia,^11^ we expected abnormalities in the Treg cell compartment. Along with a CD4^+^ T-cell lymphopenia, FOXP3^+^ T-cell numbers are reduced in the blood of patients with PHTS, but we observed normal frequency of Treg cells in the blood and colon. Treg cells have normal CTLA-4, Helios, and CD25 expression, as well as normal mitochondrial polarization, together indicating a largely functional Treg cell phenotype.

This might appear contradictory to recent studies demonstrating that homozygous loss of Pten in a *Foxp3*^*Cre*^*Pten*^*fl/fl*^ murine model leads to reduced suppressive activity of Treg cells and induction of autoimmunity.^5^, ^6^ Murine Treg cells deficient in Pten display downregulation of CD25 and Foxp3 expression, impaired mitochondrial fitness, and loss of Treg cell lineage stability.^5^, ^6^ The differences in immune phenotype between mice with Treg cell–specific Pten homozygous deletion and patients with PHTS can be explained by the gene-dose effect of PTEN expression and the complementary phosphatase activity of PHLPP that we describe in this study.

PHLPP has been shown previously to be upregulated in murine and human Treg cells compared with conventional T cells and is indispensable for murine Treg cell suppressive function, as well as iTreg cell generation.^32^ Murine PHLPP1-deficient Treg cells are unable to protect against colitis induced by naive CD4^+^ T-cell transfer.^32^ Our data show that PHLPP acts as a crucial negative regulator of PI3K signaling in human subjects, as demonstrated by deleterious effects of PHLPP blockade on iTreg cell induction and natural Treg cell mitochondrial fitness. Larger patient groups are required to determine whether a loss-of-function polymorphism in PHLPP2 might contribute to the susceptibility of autoimmunity. PTEN and PHLPP both prevent excessive accumulation of pAKT, which explains our observation that despite PTEN haploinsufficiency, we found normal FOXP3^+^ Treg cell frequencies and no increase in expression their phosphorylated ribosomal protein S6 (pS6), a downstream marker of mTORC1 activation. PTEN and PHLPP have both been shown to display cytosolic and mitochondrial localization, in which PTEN regulates mitochondrial function and metabolism and PHLPP regulates mitochondrial Akt activity.^33^, ^34^ Therefore PTEN and PHLLP can modulate mitochondrial functions through regulation of the PI3K/mTOR axis but might have additional noncanonical, mTOR-independent effects on mitochondrial fitness through their protein phosphatase activities.

T- and B-cell dysfunctions have been described in patients with activating mutations in PI3Kp85α (encoded by PIK3R1)^35^ and PI3Kp110δ (encoded by PIK3CD).^36^ There are striking similarities in the clinical phenotype of patients with gain-of-function PI3K activity and patients with loss-of-function PTEN defects, such as immune dysregulation, lymphopenia, increased transitional B-cell counts, and reduced CD4/CD8 ratio. The cause of immune dysregulation in patients with PHTS is likely due to a combination of factors, including differential B-cell apoptosis, proliferation, and differentiation^11^; lymphopenia as a risk factor for autoimmunity^37^; potential abnormalities in the follicular T-cell compartment, as demonstrated in PTEN-deleted T cells in mice^38^; or alterations in Treg cells during inflammation or cellular stress. A relevant immunodeficiency component is exceptionally rare in patients with PTEN haploinsufficiency but more prevalent in patients with gain-of-function PI3K mutations, again indicating a gene-dose effect.

PTEN accumulation at the immunologic synapse in murine Treg cells after immune cell–expressed semaphorin-4a ligation with the Treg cell receptor neuropilin 1 was recently described.^39^ Deletion of PTEN in *Foxp3-Cre Pten*^*fl/fl*^ Treg cells leads to increased Akt phosphorylation at TCR microclusters on semaphorin-4a/neuropilin 1 interaction, indicating the importance of localized phosphatase action at the immunologic synapse. Our finding that PTEN and PHLPP accumulate at the immunologic synapse on TCR ligation suggests an active phosphatase network. The immunologic synapse comprises the central supramolecular activation complex characterized by accumulation of extracellular microvesicles and the peripheral supramolecular activation complex (characterized by ICAM-1 and lymphocyte function-associated antigen 1 accumulation interspersed with actively signaling TCR microclusters).^27^ Although large transmembrane phosphatases, such as CD45, are excluded from TCR microclusters,^26^, ^40^, ^41^ our data indicate that PTEN and PHLPP are found within the microclusters in Treg cells, where they might regulate AKT phosphorylation status at the immunologic synapse in a coordinated fashion.

A dysfunctional phosphatase network comprising PTEN and PHLPP, as well as the scaffold protein NHERF1, has been recently described in glioblastoma cells.^30^ NHERF1 is known as a mediator of polarizing cell activity in epithelial cells. We show that NHERF1 is expressed in human Treg cells, where TCR stimulation leads to accumulation at the immunologic synapse alongside PTEN and PHLPP. Similarly, a further scaffold protein in this phosphatase network, DLG (encoded by *DLG1*), has been recently described in Treg cells.^42^

In summary, our data suggest that *PTEN* haploinsufficiency in patients leads to immune dysregulation but permits a normal Treg cell phenotype *in vivo* because of the compensatory phosphatase activity of PHLPP. A network supported by the scaffold proteins allows active recruitment of the phosphatases to the plasma membrane at the site of TCR stimulation, thus providing a failsafe mechanism in this critical pathway through multiple layers of coordinated negative regulation.

**Key messages**

• PTEN haploinsufficiency leads to human immune dysfunction, but normal frequency and phenotype of CD4^+^FOXP3^+^ Treg cells are found.
• PTEN and PHLPP form a phosphatase network to maintain checkpoint control at the immunologic synapse in human Treg cells.

## Figures and Tables

**Table E1:** Characteristics of patients with PHTS, including age, sex, clinical phenotype, genotype variation, protein variation, and immune phenotype

Patient no.	Sex	Age (y)	Phenotype	Genotype variation	Protein variation	Autoimmunity	Lymphoid hyperplasia
1	Male	65	CS/BRRS	c.395G>A	p.Gly132Asp		•
2	Male	49	CS	c.800delA	p.Lys267ArgfsX9	•	
3	Female	34	CS	c.287C>G	p.Pro96Arg		
4	Male	7	CS	c.18_19delAG	p.Glu7AspfsX3		
5	Female	40	CS	c.1003C>T	p.Arg335X	•	
6	Female	60	CS	c.1033C>G	p.Leu345Val		•
7	Female	49	CS	c.49C>T	p.Gln17X	•	•
8	Female	60	CS	c.697C>T	p.Arg233X		
9	Male	25	CS	c.1-?_1212+?del			•
10	Female	14	CS	c.1003C>T	p.Arg335X		
11	Male	48	CS	c.388C>T	p.Arg130X	•	•
12	Female	68	CS	c.126_127insTC	p.Glu43SerfsX12		
13	Female	59	CS	c.1-?_1212+?del		•	
14	Female	50	CS	c.235G>A	p.Ala79Thr	•	
15	Male	5	CS	c.737C>T	p.Pro246Leu		
16	Male	26	CS	c.382A>G	p.Lys128Glu	•	
17	Male	40	CS	c.49C>T	p.Gln17X		
18	Male	17	CS	c.49C>T	p.Gln17X		
19	Female	14	CS	c.511C>T	p.Gln171X		
20	Male	8	CS/BRRS	c.420_421insA	p.His141ThrfsX39		
21	Male	43	CS/BRRS	c.441_442delGGinsA	p.Ala148HisfsX5		
22	Female	64	CS	c.386G>A	p.Gly129Glu		
23	Female	47	CS	c.401T>C	p.Met134Thr	•	
24	Male	6	CS	c.388C>T	p.Arg130X	•	
25	Male	14	CS	c.208C>G	p.Leu70Val	•	
26	Male	13	ASD	c.3G>T	p.Met1Ile		
27	Male	12	ASD	c.3G>T	p.Met1Ile		
28	Female	4	ASD	c.3G>T	p.Met1Ile		
29	Female	37	ASD	c.3G>T	p.Met1Ile		
30	Male	8	CS/BRRS	c.1003C>T	p.Arg335X		
31	Female	40	CS	c.751_752delGG	p.Gly251X	•	
32	Female	51	CS	c.592_601del	p.Met198LysfsX19		•
33	Male	39	CS/LD	c.634+2T>C		•	•
34	Male	9	CS	c.491delA	p.Lys164ArgfsX2		•
35	Male	15	CS	c.26delT	p.Ser10AlafsX14		•
36	Female	21	CS	c.512_513insA	p.Arg172GlufsX8		•
37	Male	39	CS/BRRS	c.376G>C	p.Ala126Pro		•
38	Female	50	CS	c.389G>A	p.Arg130Gln		•
39	Male	56	CS	c.80-?_1212+?del			
40	Female	53	CS	c.323T>C	p.Leu108Pro		
41	Female	62	CS/LD	c.1027-2A>G			
42	Male	12	BRRS	c.389G>A	p.Arg130Gln		
43	Female	31	CS	c.445C>T	p.Gln149X	•	•
44	Female	39	CS	c.388C>T	p.Arg130X		
45	Female	13	CS	c.388C>T	p.Arg130X		
46	Male	41	CS	c.1027-1G>A			
47	Male	8	BRRS	c.388C>T	p.Arg130X		
48	Female	40	BRRS	c.388C>T	p.Arg130X		
49	Male	6	BRRS	c.388C>T	p.Arg130X		
50	Female	41	CS/LD	c.765dupA	p.Glu256ArgfsX42		
51	Female	67	CS	c.106G>T	p.Gly36X		
52	Female	14	CS	c.389G>A	p.Arg130Gln		
53	Male	50	LD	c.388C>T	p.Arg130X		•
54	Female	10	BRRS	c.930_931delTA	p.Asp310GlufsX2		
55	Male	48	BRRS	c.930_931delTA	p.Asp310GlufsX2	•	•
56	Male	58	CS/LD	c.534T>G	p.Tyr178X		
57	Female	49	CS	c.493-?_1212+?del		•	
58	Male	48	CS	c.593T>A	p.Met198Lys	•	
59	Female	31	CS	c.73_74delTT	p.Leu25AspfsX17	•	
60	Male	5	BRRS	c.445C>T	p.Gln149X	•	
61	Male	50	BRRS	c.1008C>G	p.Tyr337X		
62	Male	7	BRRS	c.540C>A	p.Tyr180X		
63	Female	24	CS	c.264T>A	p.Tyr88X	•	
64	Female	30	CS	c.491delA	p.Lys164ArgfsX3	•	
65	Female	51	CS	c.802-2A>G			
66	Female	30	CS	c.445C>T	p.Gln149X		
67	Female	16	CS	c.80-?_164+?del			
68	Female	16	CS	c.1008C>G	p.Tyr337X		
69	Male	19	BRRS	c.389G>A	p.Arg130Gln		•
70	Male	5	unclassified	c.80-?_1212+?del			•
71	Male	58	CS/LD	c.534T>G	p.Tyr178X	•	•
72	Female	68	CS	c.334C>G	p.Leu112_Lys164del		•
73	Male	4	BRRS	c.404T>A	p.Ile135Lys		
74	Female	37	CS	c.1-?_164+?del			
75	Female	39	CS	c.141delG	p.Asn48ThrfsX6		
76	Female	59	CS	c.1-?_164+?del			
77	Male	36	CS	c.654C>A	p.Cys218X		
78	Male	47	CS	c.176C>G	p.Ser59X		
79	Male	65	CS	c.640C>T	p.Gln214X		

*BRRS*, Bannayan-Riley-Ruvalcaba syndrome; *CS*, Cowden syndrome; *LD*, Lhermitte-Duclos disease.

**Table E2:** Characteristics of the total PHTS group and subgroups that have been used to determine hematology and lymphocyte subset analysis

Patient group	No.	Age (mean ± SD)
**Patients with PHTS, all**‡	79	34.05 ± 20.00
Female/male sex	40/39	
**Adult subgroup for hematology analysis**∗		
Patients with PHTS	51	34.43 ± 21.32
Female/male sex	22/29	
Control subjects	235	38.86 ± 13.31
Female/male sex	132/103	
**Adult subgroup for lymphocyte subset analysis**†		
Patients with PHTS	12	45.00 ± 14.36
Female/male sex	6/6	
Control subjects	19	41.95 ± 11.90
Female/male sex	12/7	

∗ Data are presented in Fig 1, *C*.

† Data are presented in Fig 1, *E*.

‡ Patients originated from the community and tertiary referral centers of North America and Europe and were partially recruited through cohorts that have been previously described (Heindl et al,^E1^ Tan et al,^E10^ and Heald et al^E11^). In a subgroup of patients, peripheral blood or archived formalin-fixed, paraffin-embedded gastrointestinal tissue was analyzed. Endoscopy was performed as part of routine clinical procedures. Healthy blood donors with similar age and sex distribution served as blood count control subjects (n = 235). Similarly, healthy control subjects served as FACS controls for peripheral blood lymphocyte subsets.

## Methods

### Immunohistochemistry and fluorescence microscopy

The following antibodies were used: anti-CD3 (polyclonal; Dako, Glostrup, Denmark), anti-CD10 (clone 56C6; Leica, Wetzlar, Germany), anti-CD20 (clone L26; Dako), anti–Ki-67 (clone Mib-1 or anti–Ki-67–FITC clone Mib-1 FITC labeled; Dako), anti-FOXP3 (clone 236A/E7; Abcam), anti–p(Ser235/236)-S6 (clone D57.2.2E; Cell Signaling), anti-NHERF1 (NB300-536; Novus Biologicals, Littleton, Colo), and anti-PHLPP (IHC-00382; Bethyl Laboratories, Montgomery, Tex). POD-labeled anti-mouse IgG (polyclonal donkey), anti-rabbit IgG (donkey polyclonal), and anti-FITC (clone 1F8-1E4; all from Jackson ImmunoResearch) were used to detect primary antibodies. 4′, 6′-Diamidino-2-phenylindole staining was performed to visualize nuclei.

### Flow cytometry

After erythrocyte lysis (FACS Lysing Solution; BD), patient and control samples were washed with PBS, stained, and fixed with 1% formaldehyde solution. Flow cytometry was performed on a FACSCalibur (BD) by using CellQuest Pro Software (BD), as well as a FACSCanto II with BD FACSDiva software (version 6.1.3) and FACSCalibur with CellQuest Pro software (version 4.0.2), respectively. The following antibodies were used: anti-CD3 (clone SK7 or clone UCHT1, peridinin-chlorophyll-protein complex [PerCP] or Pacific Blue; BD), anti-CD4 (clone 13B8.2, allophycocyanin [APC] or phycoerythrin [PE]-cyanin 5.1; Immunotec, Vaudreuil-Dorion, Quebec, Canada), anti-CD8 (clone SK1, PerCP; BD), anti-CD25 (clone 2A3, FITC; BD), anti-CD45 (clone 2D1, APC-H7 or APC-Cy7; BD), anti-CD45RO (UCHL1, PerCP-Cy5.5; BD), anti-CD56 (clone NCAM16.2, PE-Cy7; BD), anti-CD62L (clone GREG-56, APC-Cy7; Biozol, Eching, Germany), anti-CD127 (clone R34.34, PE; Immunotec), anti-CCR7 (clone 3D12, PE-Cy7; BD), anti-FOXP3 (clone PCH101, PE; NatuTec, Frankfurt, Germany), and anti-PTEN (clone Y184; Epitomics). Isotype-matched mAbs served as controls. Treg cells in peripheral blood were detected by means of intracellular staining of FOXP3 according to the manufacturer's instructions (FOXP3 Staining Set; NatuTec) and/or expression of the surface molecule CD127 in combination with CD25 and CD4.

### Characterizing cellular levels of PTEN

PBMCs were rested for 24 hours (unstimulated) or stimulated with Dynabeads Human T-Activator CD3/CD28 (Life Technologies, Grand Island, NY; 1 bead: 2 cells) and IL-2 (100 IU/mL). Cells were then stained with CD3, CD4, CD25, and CD45RA and fixed and permeabilized with Cytofix/Cytoperm (BD Biosciences), followed by anti-PTEN staining (clone Y184; Epitomics) in BD Perm/Wash buffer, washed, and subjected to another fixation/permeabilization procedure by using the Foxp3/Transcription Factor Staining Buffer Set (eBioscience), followed by FOXP3 staining. Cells from each source were rested for 24 hours in 10% FCS RPMI or stimulated with beads (bead/cell ratio, 1:1) and IL-2 (100 IU/mL) before FACS analysis to compare PTEN levels in T cells from a patient with PHTS versus a healthy control subject.

### *In vitro* Treg cell inducer assays

Naive human T cells were cultured for 5 days with TCR stimulation (T-cell activator beads), 50 IU/mL IL-2, 5 ng/mL TGF-β, and 50 μmol/L β-mercaptoethanol in RPMI containing 5% FCS and 25 mmol/L HEPES. Biochemical inhibition of the phosphatases PTEN, PHLPP, SHIP1, and PP2A was achieved by using inhibitors with specific activity against PTEN (2 μmol/L, SF1670; Cellagentech, San Diego, Calif), PHLPP (50 μmol/L, 1,3-[[4-(2,4-diamino-5-methylphenyl)-diazenylphenyl]hydrazinylidene]-6-oxocyclohexa-1,4-diene-1-carboxy-lic acid [US NCI, NCS 45586]), SHIP1 (10 μmol/L, 3α-aminocholestane; Merck Millipore, Billerica, Mass), SHIP2 (0.57 μmol/L, AS1938909; Merck Millipore), and PP2A (0.1 nmol/L Okadaic acid; Cell Signaling), respectively. Inhibitors were used at their respective IC_50_ concentrations, except for the PHLPP inhibitor, which was used at 50 μmol/L (IC_50_, 70 μmol/L) because of the low solubility of the compound. Inhibitors were added to culture medium 2 hours after setting up iTreg cell cultures and replenished after 48 hours. Cells were analyzed by using FACS for expression of CD4, CD25, and FOXP3. Concentration-matched dimethyl sulfoxide for the highest solvent concentration was used as a control.

### Quantitative RT-PCR

Total PBMCs, total CD4^+^ T cells, CD4^+^CD25^+^CD127^low^ Treg cells, CD4^+^CD45RO^high^ Tmem cells, and CD19^+^ B cells were FACS sorted, and total cellular RNA was extracted with an RNeasy RNA extraction kit (Qiagen, Hilden, Germany). Subsequently, RNA was reverse transcribed into cDNA by using the Quantiscript Reverse Transcription kit (Qiagen). Quantitative PCR was performed with gene-specific primers (QuantiTect Primer Assays, Qiagen) against human PTEN, SHIP1, PHLPP1, PHLPP2, NHERF1, ezrin, DLG1, moesin, and FOXP3 by using the Maxima SYBR Green qPCR Master Mix (Thermo Scientific). Gene levels were normalized to 18S rRNA.

### Terminal deoxynucleotidyl transferase dUTP nick end-labeling

Apoptosis was detected by using the ApopTag Peroxidase *In Situ* Apoptosis Detection Kit (Chemicon, Temecula, Calif), as previously described.^E1^ In brief, staining for CD3, CD10, or CD20 was performed by using tyramide amplification, which created covalent linkage between fluorescent dyes and tissue components. The sections were subsequently treated with proteinase K. After blockade of the remaining peroxidase activity, the terminal deoxynucleotidyl transferase dUTP nick end-labeling assay was performed with anti-digoxigenin–POD and subsequent tyramide fluorescence probe amplification. Counterstaining was performed with 4′, 6′-diamidino-2-phenylindole.

### T-cell proliferation suppression assay

iTreg cells were labeled with the CellTrace Violet Cell Proliferation Kit (Life Technologies) and cocultured with carboxyfluorescein succinimidyl ester–labeled allogeneic CD4^+^ naive (responder) T cells at a 1:1 ratio. Cells were cultured for 4 days before being subjected to FACS analysis to determine cell proliferation based on carboxyfluorescein succinimidyl ester dilution. The proliferation of responder T cells without the presence of iTreg cells was used as a control.

### *In vitro* apoptosis assay

PBMCs were stimulated with PHA (1.25 μg/mL) and IL-2 (100 IU/mL) for 3 to 5 days and for another 2 to 5 days with IL-2 alone (100 IU/mL). T-cell blasts were subsequently stimulated with increasing concentrations of recombinant human CD95L and enhancer (Alexis Biochemicals, Lausen, Switzerland) for 24 hours. After staining with Annexin V and propidium iodide, apoptosis was quantified by using FACS. Titration curves and control values from healthy donors served as control values.

### Immunoblotting and immunoprecipitation

NIH/3T3 cells were lysed in protein lysis buffer. For immunoprecipitation, protein lysates were incubated with goat polyclonal anti-human PTEN antibody (N-19; Santa Cruz Biotechnology, Dallas, Tex) and protein G–Sepharose (GE Healthcare Life Sciences, Fairfield, Conn) overnight at 4°C. Immunoprecipitates were washed in lysis buffer and then denatured in an equal volume of 2× SDS-PAGE sample buffer and subjected to electrophoresis for immunoblotting. For Western blotting, whole-cell lysates were mixed with the same volume of 2× SDS-PAGE sample buffer and subjected to electrophoresis for immunoblotting. The primary antibodies used for immunoblotting were rabbit polyclonal anti-human PHLPP (IHC-00382; Bethyl Laboratories), rabbit polyclonal anti-human NHERF1 (EBP50; Novus Biologicals), and goat polyclonal anti-human PTEN (N-19; Santa Cruz Biotechnology). Western blotting was carried out according to the standard protocol.

### PTEN mutation and PHLPP2 polymorphism analysis

Genomic DNA was extracted from peripheral blood leukocytes by using standard protocols. PTEN mutation analysis with Sanger sequencing and copy number analysis with multiplex ligation-dependent probe amplification were performed by using a combination of different standard methods.E1, E2, E3, E4, E5 The PHLPP2 L1016S polymorphism (rs61733127) was analyzed by using LightScanner High Resolution DNA Melting (HRM) analysis. Primers and probe were designed to have a melting temperature of between 59°C and 65°C with LightScanner Primer Design software (Idaho Technology, Salt Lake City, Utah). Oligonucleotide sequences were as follows: PHLPP2, GCTGTCAATGCTGTACGTC (forward); PHLPP2, ATATTCAAATAAACTACCATCGCCC (reverse); and TGTGCACATCAGCGCAGAG-phosphate (probe). PCR reactions were performed at an optimized annealing temperature of 62.5°C in a total volume of 10 μL containing 4 μL of 2.5× LightScanner Mastermix, 1 μL of genomic DNA (25 ng/μL), 1 μL of primer mix (final concentration: 0.1 μmol/L of forward primer, 0.5 μmol/L of reverse primer, and 0.5 μmol/L of the probe), and 4 μL of LightScanner Reagent Grade Water. Reaction mixtures were overlaid with 20 μL of mineral oil to avoid evaporative losses, ensuring melting curve uniformity. HRM curves were analyzed by using the LightScanner software (Idaho Technology), according to the protocols described previously.^E6^ The “unlabeled probe” module in the “genotyping” mode of the software was used for HRM analyses involving negative filter, normalization, and grouping. Genotyping by using HRM was based on probe melting temperature, which was obtained from negative derivative melting curve plots. These analytic methods have been used previously for single nucleotide polymorphism genotyping.E7, E8 Samples showing abnormal melting curves were purified and sent for Sanger sequencing for validation. Sequences were analyzed by using Mutation Surveyor DNA Variant Analysis Software (SoftGenetics, State College, Pa).
